# Academic Career Exploration: Learner Opportunities Through the Office of Faculty Affairs

**DOI:** 10.15766/mep_2374-8265.11460

**Published:** 2024-10-31

**Authors:** Raymond Lucas, Nicholas Brutus, Guadalupe Federico-Martinez, Chloe Soremekun, Mekbib Gemeda, Jose Rodriguez, Janet Townsend, Edward Callahan, John Paul Sánchez

**Affiliations:** 1 Interim Senior Associate Dean for The George Washington University Regional Medical Campus and Professor of Emergency Medicine, The George Washington University School of Medicine and Health Sciences; 2 First-Year Resident, Department of Urology, Yale School of Medicine; 3 Assistant Dean, Faculty Affairs and Career Development, and Associate Professor, Medicine, University of Arizona College of Medicine - Phoenix; 4 Fourth-Year Medical Student, Rowan-Virtua School of Osteopathic Medicine; 5 Vice President of Diversity and Inclusion, Eastern Virginia Medical School; 6 Associate Vice President for Health Equity, Diversity, and Inclusion, University of Utah Health; 7 Professor Emeritus of Family Medicine, Geisinger Commonwealth School of Medicine; 8 Associate Vice Chancellor Emeritus for Academic Personnel, University of California, Davis, Schools of Human Health; 9 Dean, Universidad Central Del Caribe School of Medicine

**Keywords:** Career Development, Leadership, Career Choice, Continuing Professional Development, Faculty Affairs, Faculty Development, Health Care Workforce, Leadership Development/Skills, Self-Assessment

## Abstract

**Introduction:**

Early exposure to medical school offices can help to facilitate interest in academic medicine and senior leadership positions. This workshop provides an overview of the roles, responsibilities, and activities within the Office of Faculty Affairs (OFA) and highlights opportunities for trainee engagement and leadership.

**Methods:**

The Kern model was applied in the design of a 60-minute interactive module for medical students. The module consisted of a didactic portion overviewing OFA roles and responsibilities. Learners were engaged through reflection exercises and case discussions on how trainees could develop competencies through engagement with the OFA, including contributing to faculty grievance and promotion processes. A summary sheet was created to explicitly describe faculty leadership competencies potentially developed by medical students through faculty affairs–related activities.

**Results:**

The module was presented at five conferences, and 45 participants responded to the workshop evaluation forms. A comparison of pre- and postworkshop responses showed a statistically significant increase in perceived knowledge in identifying leadership opportunities for trainees to become engaged through the OFA and in listing skills that are important for an OFA dean.

**Discussion:**

Medical trainees not only can benefit from gaining awareness of opportunities for engagement through the OFA but also have the ability to gain foundational skills and competencies to eventually serve as OFA leaders. This workshop provides an early exposure to the OFA for trainees and an opportunity to realize a potential career in academic medicine beyond the faculty role.

## Educational Objectives

By the end of this session, learners will be able to:

1. Describe the ABCs of Offices of Faculty Affairs.
2. List skills and characteristics needed to work in faculty affairs.
3. Describe learner involvement in faculty affairs.

## Introduction

Academic medicine is moving towards diversifying the academic workforce and transforming medical education to reflect the needs of the United States population. The Association of American Medical Colleges’ online planning resource, Careers in Medicine, presents a four-phase career-planning process.^1^ However, the emphasis remains on self-exploration as it relates to specialty choice and stops at preparation for residency.^1^ There is a growing body of literature explaining the motivation for selecting an academic career overall (i.e., physician-investigator, clinical educator) and leadership roles such as department chair or vice-chair.^2–4^ For racial and ethnic minority students, lack of information on academic options is a barrier to considering an academic career, suggesting that early exposure is important.^5^ Nonetheless, there is a paucity of literature known to us that specifically explores the value of and charts the path to academic administration in faculty affairs or how early engagement with faculty affairs can support choosing an academic career.

*MedEdPORTAL* continues to serve as a leading journal for educators to collaborate and design novel curricula to further explain and showcase academic processes. There are limited resources that teach the needed skills to successfully navigate an academic career path as a faculty member and medical school leader in admissions, student affairs, and diversity, equity, and inclusion.^6–10^ A number of publications center on novel methods for faculty onboarding, unconscious bias in training, evolution of faculty affairs offices, and academic appointment and promotion processes.^11–14^ However, curricula on highlighting the Office of Faculty Affairs (OFA) through the lens of medical student engagement and standardized professional development have yet to be explored.

Established in 2010, Building the Next Generation of Academic Physicians (BNGAP) has championed the development of innovative curricula to recruit, retain, and promote diverse trainees in academic medicine. BNGAP designs curricula aimed to raise awareness among UME and GME trainees for academic careers. This workshop was designed by members of the BNGAP Curriculum Committee and by fellows of the National Center for Pre-Faculty Development. The workshop was a part of a curriculum entitled Engagement and Leadership in Academic Medicine for Diverse Trainees Seminar.

The baseline for designing this module was derived from a national survey of faculty leadership development programs.^15^ This national initiative highlighted the need for the development of defined leadership competencies and utilization of different approaches to teaching and program evaluation.^15^ Our workshop was modeled using Bandura's theory of self-efficacy^16^ with a goal of providing exposure, role modeling, mentoring, and observation. While student engagement with Offices of Student Affairs, Admissions, and DEI are common, introducing medical students and residents to the OFA has not been reported before and is a novel way to give them additional insights to prepare them for academic careers. This workshop incorporated an interactive component to parallel student engagement with faculty affairs to faculty competencies, thus building student self-efficacy.

## Methods

The six-step Kern approach to curriculum development was used to structure the workshop to provide medical students with familiarity regarding engagement opportunities with the OFA and future leadership roles within the OFA.^17^

The workshop engaged trainees through a PowerPoint presentation with multiple case-based discussions, and with small- and large-group reflective exercises. The workshop was developed to further explain leadership opportunities and processes within the OFA. Its broader goal was to sensitize and socialize medical students to the norms of faculty life via in-depth exploration of OFAs for their career planning. The module highlighted foundational skills and competencies acquired through direct engagement between medical students and faculty affairs leaders. The team of diverse coauthors had a range of experience in faculty affairs, either at their respective institutions or at prior institutions in their academic journey. The design of the workshop content was reflected in the experience of the working group.

Step 1, general needs assessment and problem identification, included conducting a literature search on medical student and resident awareness and engagement with the OFA. In step 2, we referenced the faculty leadership competencies developed by Lucas and colleagues^15^ and the AAMC's section of Faculty Affairs Performance Framework^18^ to outline core elements for trainees to develop through engagement in the OFA. In addition, meetings were held with medical students and residents to assess their prior knowledge and experiences with the OFA. In step 3, we determined the goals/objectives for the module based on our literature review and through discussion with the coauthors. Four of the coauthors (Raymond Lucas, Janet Townsend, Guadalupe Federico-Martinez, and Edward Callahan) had had OFA positions at different institutions. For step 4, we opted to format the content in a PowerPoint presentation that contained reflection exercises and case discussion. In step 5, the module was delivered to groups of participants at a series of institutional and national conferences targeted to medical students, residents, fellows, and junior faculty interested in leadership opportunities in academic medicine. Step 6, evaluation and feedback, was accomplished through dissemination of pre- and postworkshop evaluations to each participant.

### Implementation

The module began with dissemination of preworkshop evaluation forms (Appendix A) that allowed participants to self-identify demographic information and indicate their level of knowledge of the OFA. After dissemination of the preworkshop evaluations, a PowerPoint slide set (Appendix B) showcased the content, which included a short didactic portion detailing the professional tenets of faculty affairs (Appendix C). An interactive activity was built into the slide set to engage participants to better understand practical day-to-day activities as well as office purpose and mission. Participants were asked to engage in brief case scenarios that pertained to common scenarios within the OFA and to identify the core domains and opportunities for collaboration.

Workshop participants were prompted to reflect on prior engagements with the OFA as medical students and/or during residency. Following the reflection exercise, the facilitator discussed potential opportunities/scenarios in which medical students might be involved in the OFA and leadership competencies developed through engagement (Appendix D). Case 2 (Appendix E) highlighted an example letter of recommendation (Appendix F) written by a medical student for promotion of a faculty member to tenure. This letter could also be referred to during slides 15–16 and 19. Three case discussions with reflective question prompts near the end of the module allowed for further discussion of student engagement and OFA leadership competencies. Customized slides on the facilitators’ personal journeys and leadership roles were then shared with participants. The workshop concluded with a brief question-and-answer session, as well as dissemination of a postworkshop evaluation survey (Appendix A). The postevaluation surveys were provided in order to transform the workshop based on the suggestions in participant feedback.

This workshop could be delivered by one or two facilitators who, ideally, would have experience working with the OFA or be leaders in the OFA. Prior to the session, speakers had to dedicate at least 3 hours to reviewing the module, including the facilitator guide (Appendix G), which served to guide them through the content. To optimally deliver the module, audiovisual equipment for PowerPoint display, tables and chairs/desks arranged for small- to medium-group discussion, printed copies of the core OFA competencies summary sheet (Appendix D), cases scenarios (Appendix E), and evaluation forms (Appendix A) were required. The module could also be delivered in a virtual format and modified to the convenience of facilitators.

The workshop could be delivered in 60–75 minutes with appropriate modifications to time.

#### Timetable (60-minute presentation)

• 00:00–03:00 minutes: preassessment
• 04:00–12:00 minutes: introduction and reflection (slides 1–4)
• 13:00–30:00 minutes: PowerPoint presentation (slides 5–28)
• 31:00–45:00 minutes: case discussion (slides 29–32)
• 46:00–57:00 minutes: facilitator journey and questions (slides 33–37)
• 58:00–60:00 minutes: postassessment

This module was a component of a BNGAP Engagement and Leadership in Academic Medicine Curriculum but could also be delivered independently. Presenters were encouraged to incorporate their own experiences in the OFA and their institution's structure into the module.

## Results

This session was implemented at five conference sites (the University of Iowa Roy J. and Lucille A. Carver College of Medicine, McGovern Medical School at the University of Texas Health Science Center at Houston, University of Oklahoma College of Medicine, Weill Cornell Medicine Medical College, and the BNGAP Virtual National Pre-Faculty Development Conference) with a total of 45 participants. A total of 45 individuals completed the pre-/postworkshop evaluation forms. Across the five implementations, the workshop was implemented by five single presenters with titles in the OFA (assistant dean, associate dean, or senior associate dean).

Of the 45 participants who responded to the preconference evaluation, 35 identified as medical students, three as residents, two as fellows, and five as other health care professionals. Among the 45 respondents, two (4%) self-identified as Native American/Alaska Native; five (11%) as Asian; 12 (26%) as Black or African American; 14 (31%) as Latino/a/x, Hispanic, or of Spanish origin; 14 (31%) as White/Caucasian; and two (4%) indicated “other.” Seventeen (38%) self-identified as male and 20 (44%) as female, while eight (18%) selected “prefer not to answer” or “other” as a response. Six (13%) self-identified as gay, lesbian, or bisexual.

The pair-sampled Wilcoxon signed rank test was used to determine a statistically significant change in participants’ self-perception of individual confidence between the pre- and posttest evaluations for two specific questions. Among 36 respondents with paired responses for the question “How knowledgeable are you in identifying leadership opportunities for trainees to become engaged through Office of Faculty Affairs?”, the median improved from 0 (*Not Knowledgeable*) to 2 (*Knowledgeable*; *p* < .01). For the question “List skills important for a Faculty Affairs Dean,” with responses from 0 (*No Confidence*) to 4 (*Complete Confidence*), the median improved from 2 to 4 (*p* < .01).

Participants were asked to complete a postworkshop evaluation survey to indicate, on a Likert scale (0 = *Strongly Disagree*, 4 = *Strongly Agree*), the degree to which the six learning objectives regarding OFAs had been met. Among the 38 who responded to the question for learning objective 1, “Recognize the OFA within an institutional structure,” the mean and median values were 3.7 and 4.0, respectively. For learning objective 2, “Identify the mission, duties, and responsibilities of Faculty Affairs,” the mean and median values were 3.7 and 4.0, respectively. For learning objective 3, “Describe how the Office of Faculty Affairs assists faculty with professional advancement (the ABCs),” the mean and median values were 3.8 and 4.0, respectively. For learning objective 4, “Describe student involvement opportunities in Faculty Affairs and Development,” the mean and median values were 3.4 and 4.0, respectively. For learning objective 5, respondents were asked to rate their confidence in the ability to “List skills and characteristics needed to work in Faculty Affairs” on a scale from 0 (*No Confidence*) to 4 (*Complete Confidence*). The mean and median values were 3.6 and 4.0, respectively. For learning objective 6, “Describe tenure and promotion processes,” the mean and median values were 3.7 and 4.0, respectively. In addition, across all learning objectives, over 95% of participants from all five sites agreed that the objectives had been met.

Formal comments were collected from workshop participants to identify strengths of the content and suggestions on how to enhance the module. Generally, the participants found the workshop to be “practical,” “informative,” and “engaging” and described the content as transparent and structured. A number of participants commented on the levels of engagement, from UME to GME: “Gave me awareness of numerous ways to be involved with faculty,” “appreciated how the presenters related it back to [medical] students,” and “it felt very applicable to me at the student level.” Respondents appreciated the speakers’ expertise on the topic reflected in the structure of the presentation: “Speakers made sure to include detailed outlines of the information,” “this was an area I knew very little about beforehand, I really liked the specific shared experiences they presented,” and “provided strong motivation to pursue academic medicine.” Lastly, the participants shared their appreciation for explanations of unfamiliar processes: “Enjoyed the A.B.C.s of faculty affairs to explain a new and sometimes confusing topic,” “first time I've learned about these processes,” and “made something I didn't know about easily approachable.”

With regard to areas of improvement for the workshop, participants provided a few recommendations. The common theme of participants’ unfamiliarity with tenure arose: “Tenure was hard to follow, because I had no prior knowledge,” and “good description of tenure promotion without real explanation of what ‘tenure’ is.” Two respondents recommended the addition of concrete examples on how junior trainees or junior faculty could build their portfolio and leverage for promotion negotiations.

## Discussion

Many medical trainees may be unfamiliar with the OFA, including services offered to faculty and opportunities for learner engagement, and therefore may not see faculty affairs as a future senior leadership pursuit. This module, designed to promote awareness, presents information on the structure and function of the OFA and shares examples of trainee involvement to introduce potential career endeavors. Few students indicated a high level of expertise in familiarity with the OFA. Among all learning objectives, over 95% of participants agreed that the objectives had been met.

This curriculum was optimized for student learning through interactive reflections exercises and case discussions facilitating exploration of the duties and activities of the OFA. Attendees were able to build their familiarity with academic medicine careers through increased recognition of the roles and responsibilities of the OFA. The mnemonic of the ABCs of faculty affairs provided participants with a thorough and simple overview of the duties of the office. Appendix D helped learners appreciate how core faculty leadership competencies can be attained through engagement with the OFA.

The workshop was enhanced over its five implementations. For the second implementation, the mnemonic of ABCs of OFAs was developed to simplify delivery of the OFA duties. Additional cases focused on the grievance process and writing a letter of recommendation were added to the case on the search process. Specific slides on the tenure process were added to clarify the meaning of and process towards tenure. Facilitators are encouraged to modify the slides and highlight their own institution's structure and function or experiences with the OFA.

Future directions for OFA-related modules could include illustrating the duties and competencies of faculty affairs leadership. Mentoring, professional development, mitigation of implicit bias, and career planning might all be specific workshops dedicated to linking trainee experiences with faculty affairs leadership and practice.

Despite having an ethnically and geographically diverse array of workshop participants, the development of this module was based on a limited sample size composed of mostly medical students. The limited number of graduate trainees (i.e., residents and fellows) potentially reduces the applicability of this group. However, in the quantitative and qualitative feedback received from graduate medical trainees, there were no comments on the workshop not being received favorably by this audience. If this module were to be delivered exclusively to a resident and fellow group, then facilitators should consider adapting the content to focus on GME experiences and opportunities. More data are needed for better content delivery to GME trainees; however, the learning objectives are relevant to both UME and GME trainees.

There were also limitations in the assessment of participant confidence levels. While this module was designed to assess immediate pre-post self-perceived confidence levels of learning objectives, long-term retention was not assessed. The scope and duration of the module were a limitation for evaluating more complex outcomes, for example, sustained participant self-efficacy. A 6-month or 12-month reassessment of participants’ perception of the content may help to showcase sustained effectiveness of the workshop.

Medical schools interested in increasing student self-efficacy towards academic career choice can offer this module in addition to other *MedEdPORTAL* publications^8,10^ on engaging with other medical school offices. As medical trainees consider a career in academic medicine, this module can serve as an introduction to opportunities to engage with the OFA. Importantly, this workshop raises awareness of the roles and responsibilities within the OFA, through a student engagement lens, which ultimately allows trainees to perceive growth in foundation skills and leadership competencies.

## Appendices


Evaluation.docxOFA and Learner Engagement.pptxThe Value of FA and FD Offices.docxActivity Sheet.docxCase Discussion.docxExample Letter of Recommendation.docxFacilitator Guide.docx
*All appendices are peer reviewed as integral parts of the Original Publication.*
