# Supplementary material for: Academic Career Exploration: Learner Opportunities Through the Office of Faculty Affairs
Source: MedEdPORTAL. 2024 Oct 31;20:11460. doi: 10.15766/mep_2374-8265.11460 (PMC11525038; doi:10.15766/mep_2374-8265.11460)
Supplement: Supplementary file 1 — Evaluation.docxOFA and Learner Engagement.pptxThe Value of FA and FD Offices.docxActivity Sheet.docxCase Discussion.docxExample Letter of Recommendation.docxFacilitator Guide.docx [file mep_2374-8265.11460-s001.zip › A. Evaluation.docx]

These pre- and post-assessment surveys are to be distributed before and after implementation of the module.

**Office of Faculty Affairs**

**Engagement and Leadership Opportunities for Learners**

Pre-Assessment

| Unique Identifier |  | | | | | | | |
| --- | --- | --- | --- | --- | --- | --- | --- | --- |
| How knowledgeable are you in identifying leadership opportunities for trainees to become engaged through Office of Faculty Affairs | **Not Knowledgeable** | | **Somewhat Knowledgeable** | | **Knowledgeable** | | **Very knowledgeable** | |
| How much CONFIDENCE do you have in your ability to…  List skills important for a Faculty Affairs Dean | **No Confidence**  **0** | **1** | | **2** | | **3** | | **Complete**  **Confidence**  **4** |
| DEMOGRAPHICS: | | | | | | | | |
| In which STATE is your medical school or residency program located? _____________________ | | | | | | | | |
| Are you a (circle one):   1. Medical Student 2. Intern/Resident 3. Fellow 4. Other (please specify):_______________________________ | | | | | | | | |
| What is your race/ethnicity (circle all that apply)?   1. American Indian or Alaska Native 2. Native Hawaiian or Other Pacific Islander 3. Asian 4. Black or African-American 5. Latina/o/x/e, Hispanic or of Spanish Origin+ (LHS+) 6. White 7. Other (please specify): | | | | | | | | |
| How do you self-identify (circle one)?   1. Straight or Heterosexual 2. Gay or Lesbian 3. Bisexual 4. Other (please specify): ____________________ | | | | | | | | |
| How do you self-identify? Note: Respondents who self-identify as “Transgender female-to-male,” “Transgender male-to-female,” or “Transgender do not identify as exclusively male or female” are combined and displayed as “Transgender” (circle all that apply):   1. Male 2. Female 3. Transgender 4. Other: ________________________________ | | | | | | | | |

**Post-Assessment**

| Unique Identifier |  | | | | | | | | | | | |
| --- | --- | --- | --- | --- | --- | --- | --- | --- | --- | --- | --- | --- |
| How knowledgeable are you in identifying leadership opportunities for trainees to become engaged through Office of Faculty Affairs | | | **Not Knowledgeable** | | | **Somewhat Knowledgeable** | | **Knowledgeable** | | | **Very knowledgeable** | |
| How much CONFIDENCE do you have in your ability to…  List skills important for a Faculty Affairs Dean | | | **No Confidence**  **0** | | **1** | | **2** | | **3** | | | **Complete**  **Confidence**  **4** |
| To what EXTENT do you agree that the workshop learning objectives were met | **Strongly agree** | **Agree** | | **Neither agree or disagree** | | | **Disagree** | | | **Strongly disagree** | | |
| Describe the ABCs of Offices of Faculty Affairs | SA | A | | N | | | D | | | SD | | |
| List skills and characteristics needed to work in faculty affairs | SA | A | | N | | | D | | | SD | | |
| Describe learner involvement in faculty affairs | SA | A | | N | | | D | | | SD | | |
| What did you like about this workshop? | | | | | | | | | | | | |
| What suggestions do you have to improve this workshop? | | | | | | | | | | | | |
