# Supplementary material for: Academic Career Exploration: Learner Opportunities Through the Office of Faculty Affairs
Source: MedEdPORTAL. 2024 Oct 31;20:11460. doi: 10.15766/mep_2374-8265.11460 (PMC11525038; doi:10.15766/mep_2374-8265.11460)
Supplement: Supplementary file 1 — Evaluation.docxOFA and Learner Engagement.pptxThe Value of FA and FD Offices.docxActivity Sheet.docxCase Discussion.docxExample Letter of Recommendation.docxFacilitator Guide.docx [file mep_2374-8265.11460-s001.zip › B. OFA and Learner Engagement.pptx]

## Slide 1
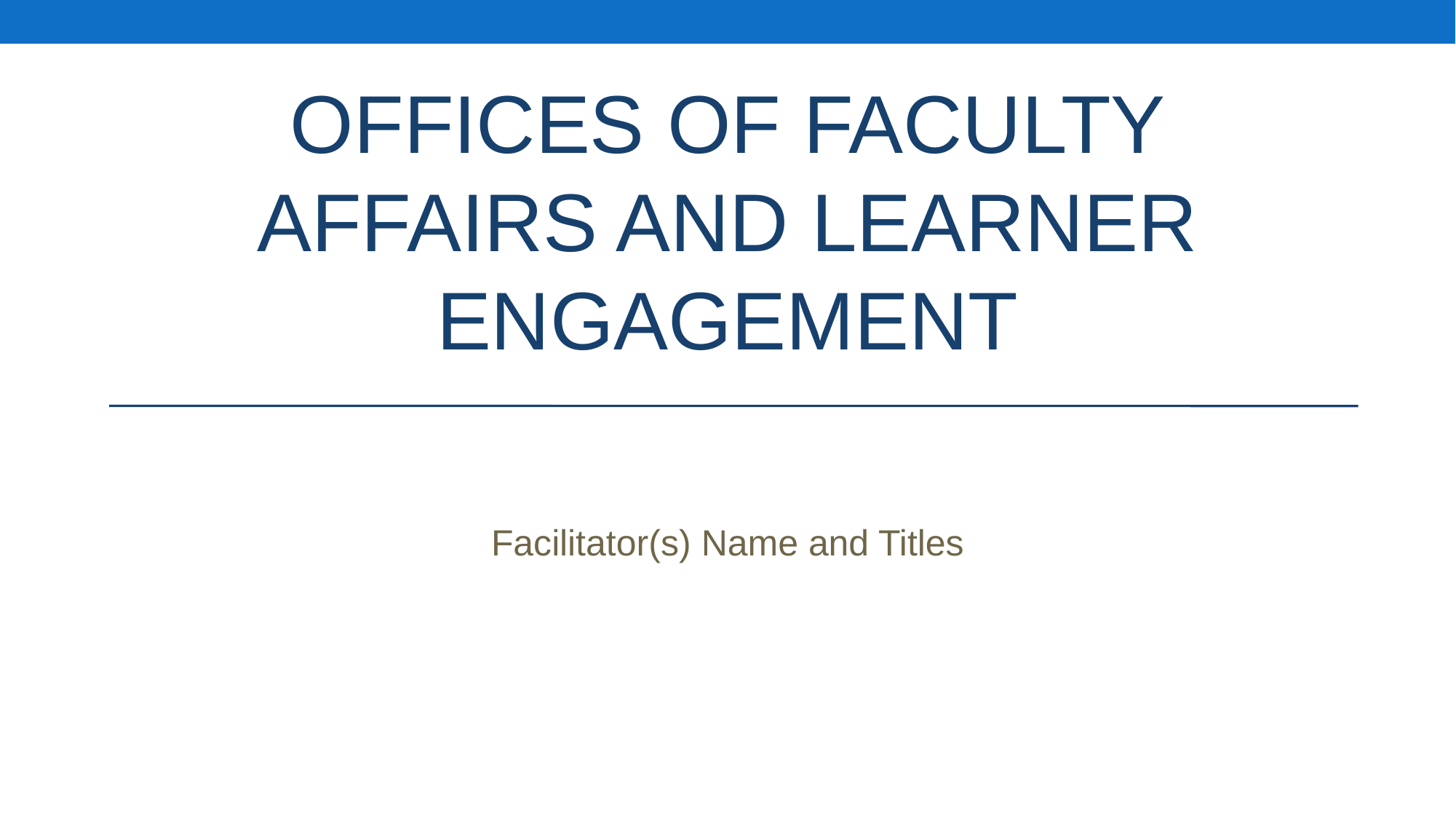

# OFFICES OF FACULTY AFFAIRS AND LEARNER ENGAGEMENT
Facilitator(s) Name and Titles

## Slide 2
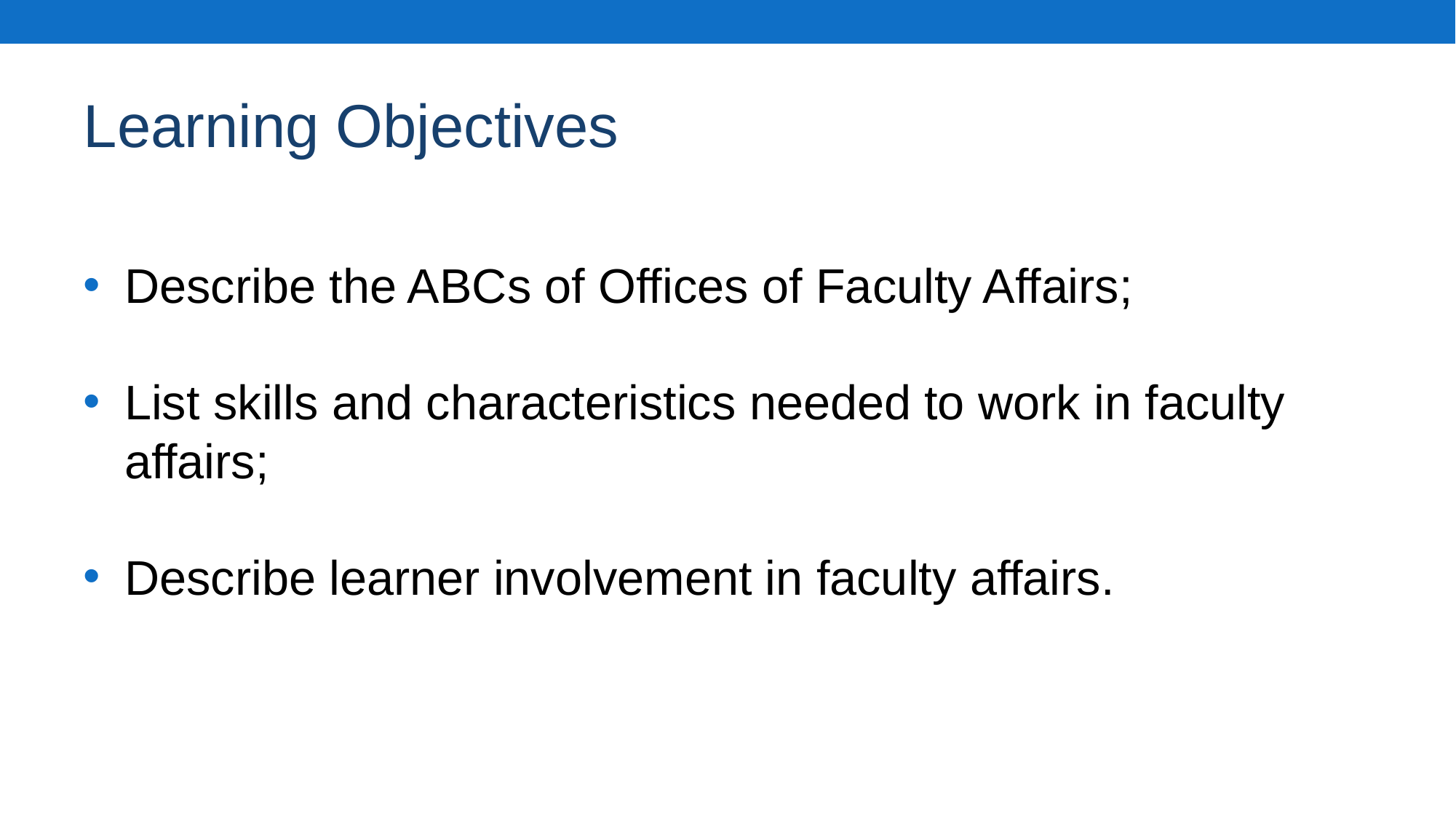

# Learning Objectives
Describe the ABCs of Offices of Faculty Affairs;
List skills and characteristics needed to work in faculty affairs;
Describe learner involvement in faculty affairs.

## Slide 3
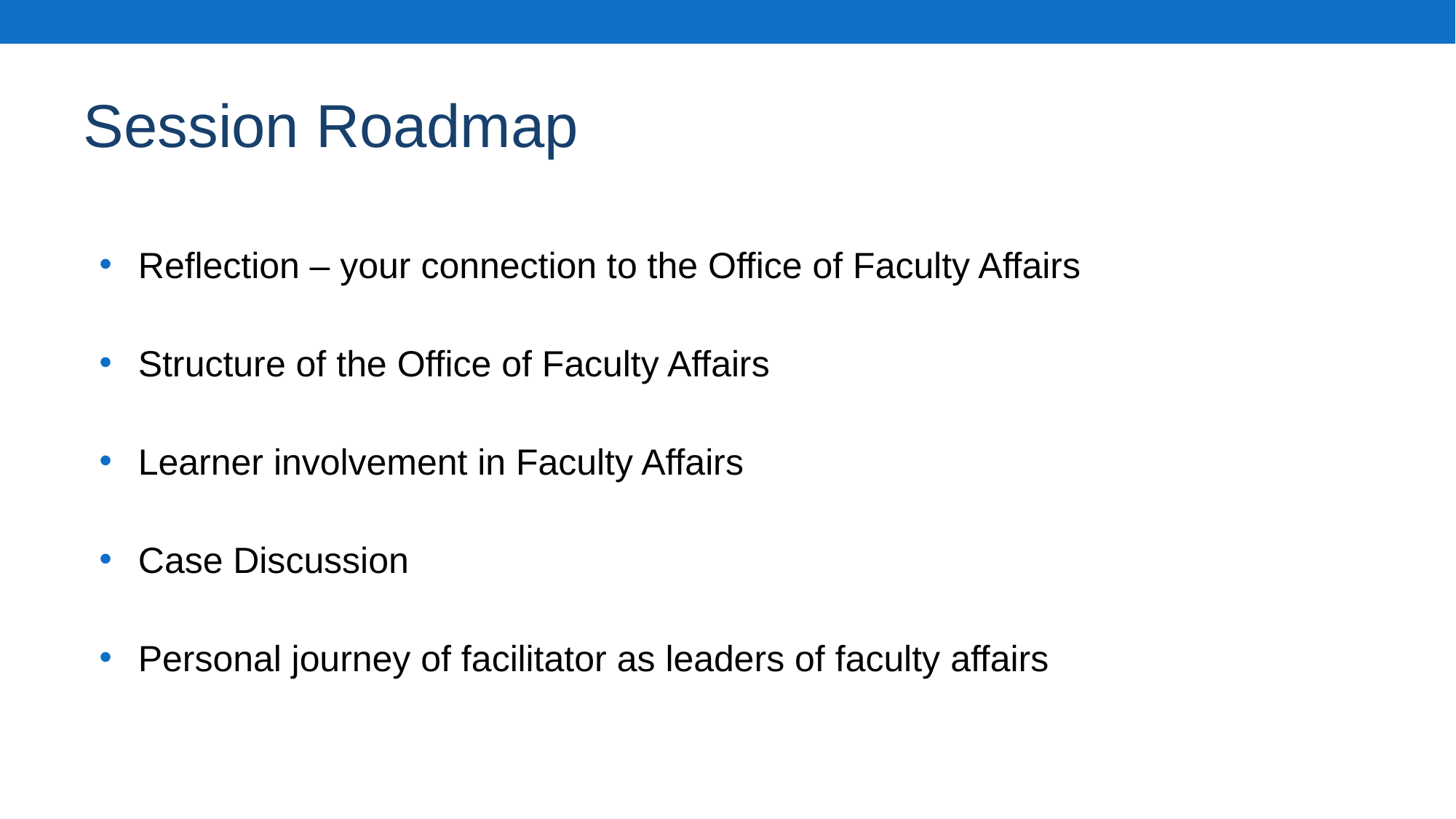

# Session Roadmap
Reflection – your connection to the Office of Faculty Affairs
Structure of the Office of Faculty Affairs
Learner involvement in Faculty Affairs
Case Discussion
Personal journey of facilitator as leaders of faculty affairs

## Slide 4
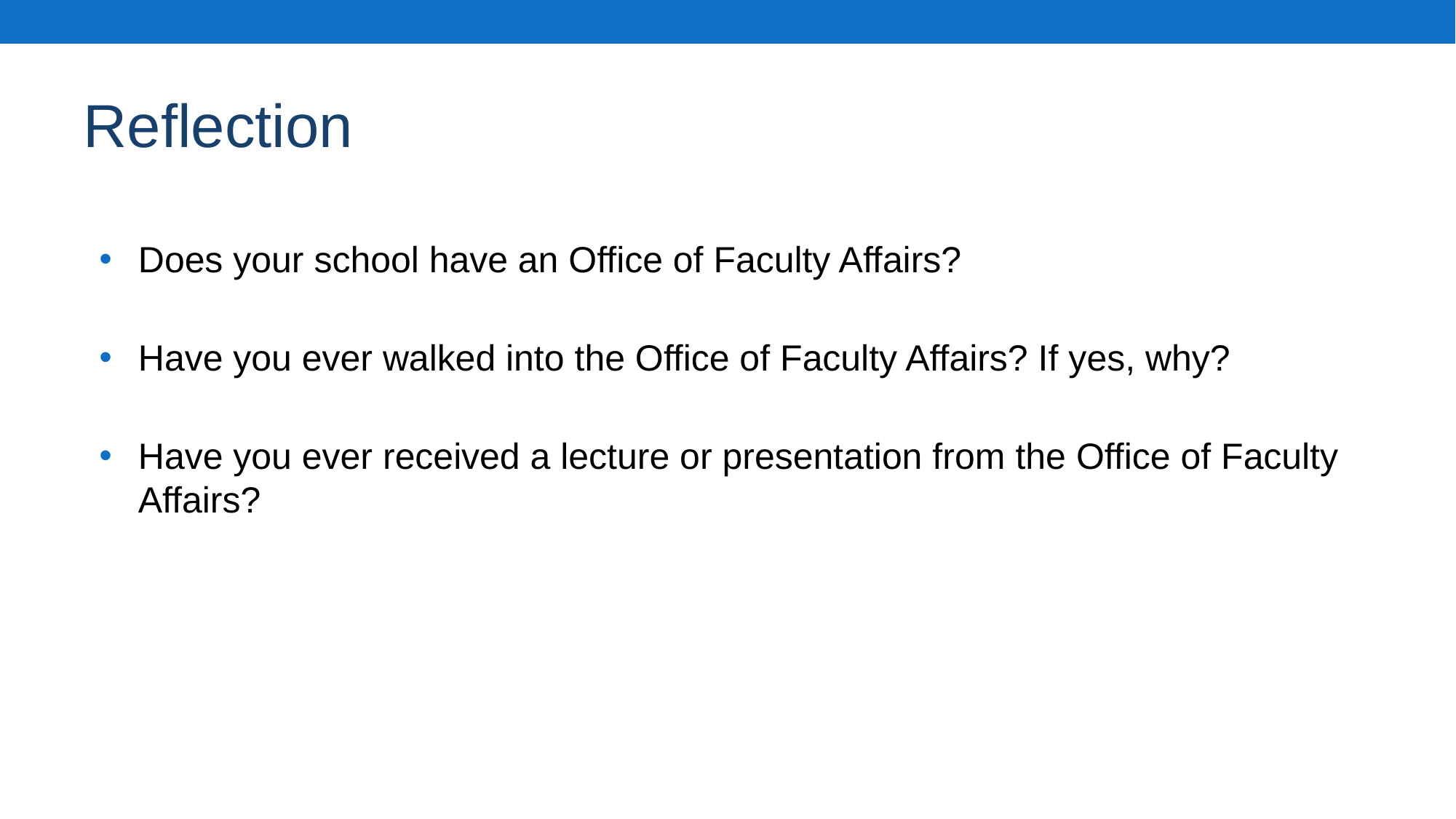

# Reflection
Does your school have an Office of Faculty Affairs?
Have you ever walked into the Office of Faculty Affairs? If yes, why?
Have you ever received a lecture or presentation from the Office of Faculty Affairs?

## Slide 5
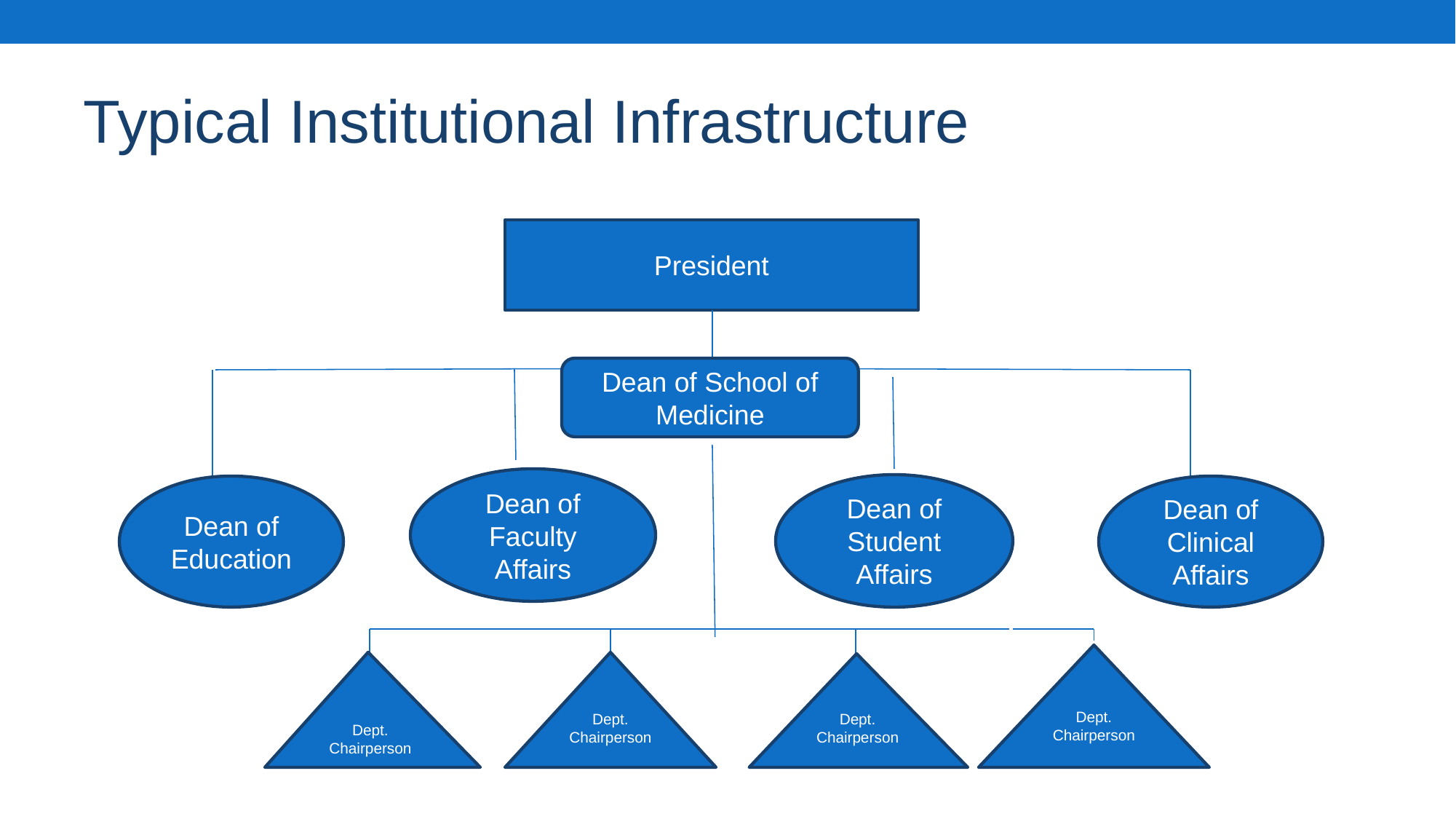

# Typical Institutional Infrastructure
President
Dean of School of Medicine
Dean of Faculty Affairs
Dean of Student Affairs
Dean of Education
Dean of Clinical Affairs
Dept. Chairperson
Dept. Chairperson
Dept. Chairperson
Dept. Chairperson

## Slide 6
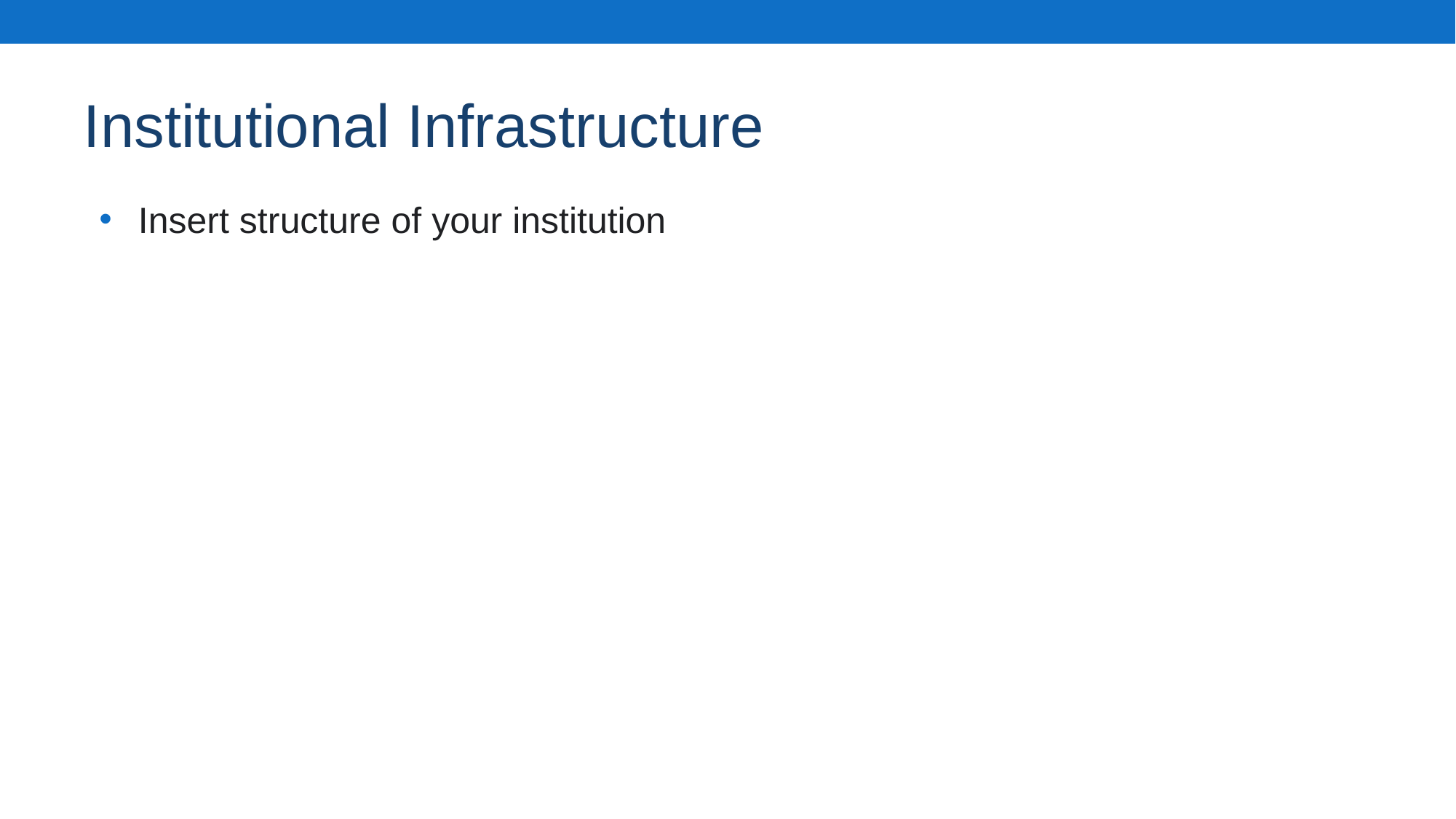

# Institutional Infrastructure
Insert structure of your institution

## Slide 7
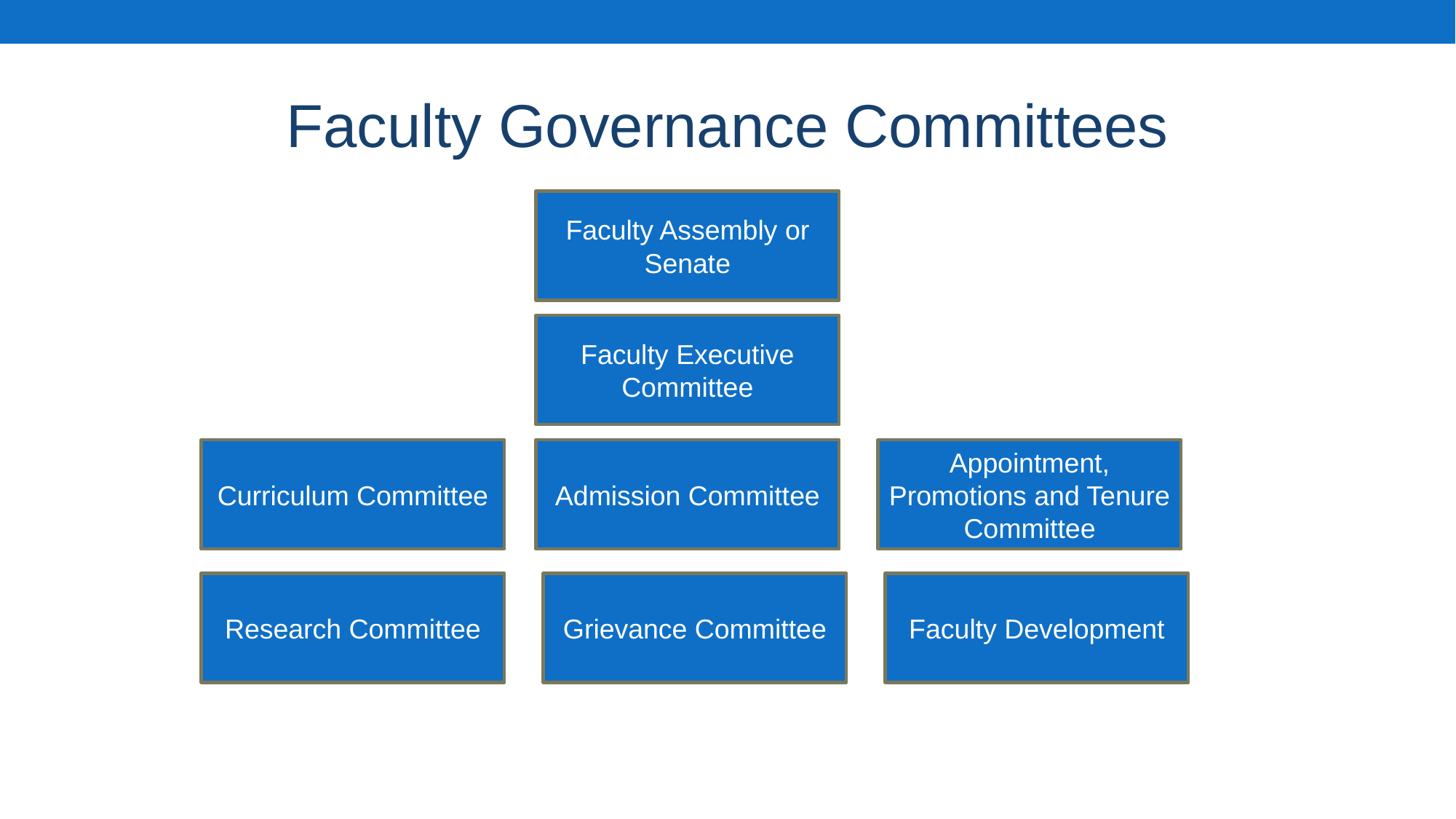

# Faculty Governance Committees
Faculty Assembly or Senate
Faculty Executive Committee
Curriculum Committee
Admission Committee
Appointment, Promotions and Tenure Committee
Research Committee
Grievance Committee
Faculty Development

## Slide 8
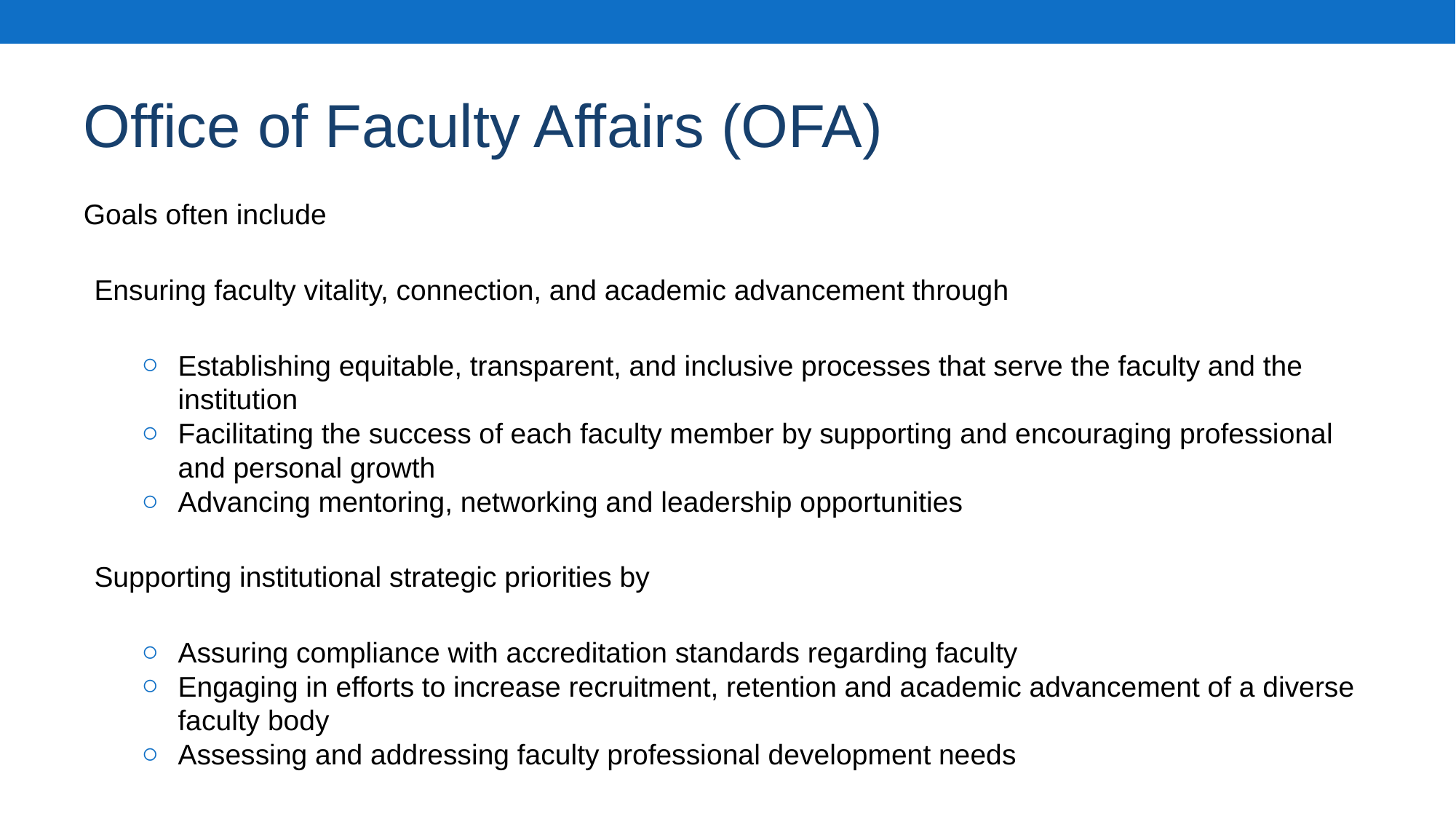

# Office of Faculty Affairs (OFA)
Goals often include
Ensuring faculty vitality, connection, and academic advancement through
Establishing equitable, transparent, and inclusive processes that serve the faculty and the institution
Facilitating the success of each faculty member by supporting and encouraging professional and personal growth
Advancing mentoring, networking and leadership opportunities
Supporting institutional strategic priorities by
Assuring compliance with accreditation standards regarding faculty
Engaging in efforts to increase recruitment, retention and academic advancement of a diverse faculty body
Assessing and addressing faculty professional development needs

## Slide 9
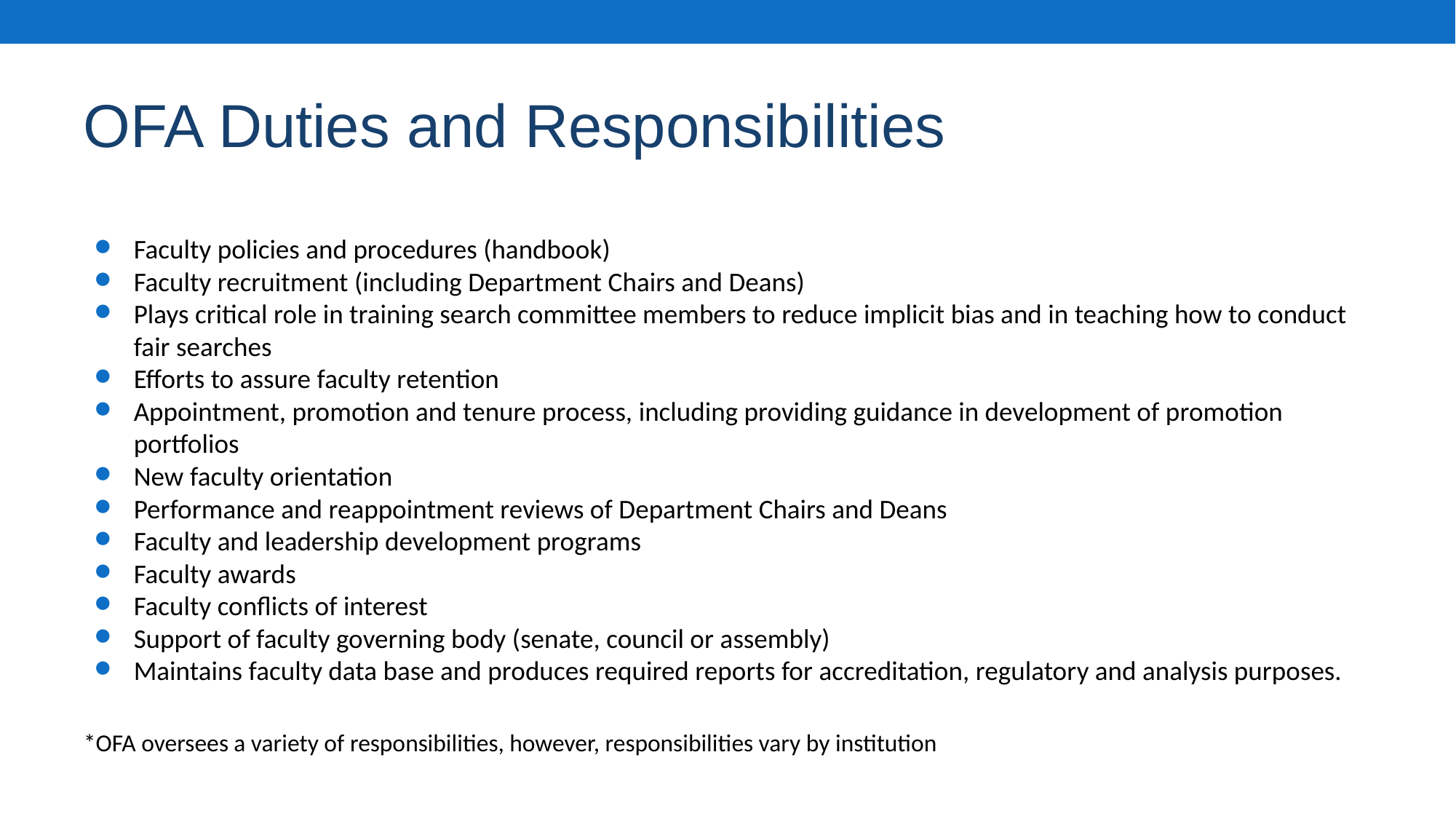

# OFA Duties and Responsibilities
Faculty policies and procedures (handbook)
Faculty recruitment (including Department Chairs and Deans)
Plays critical role in training search committee members to reduce implicit bias and in teaching how to conduct fair searches
Efforts to assure faculty retention
Appointment, promotion and tenure process, including providing guidance in development of promotion portfolios
New faculty orientation
Performance and reappointment reviews of Department Chairs and Deans
Faculty and leadership development programs
Faculty awards
Faculty conflicts of interest
Support of faculty governing body (senate, council or assembly)
Maintains faculty data base and produces required reports for accreditation, regulatory and analysis purposes.
*OFA oversees a variety of responsibilities, however, responsibilities vary by institution

## Slide 10
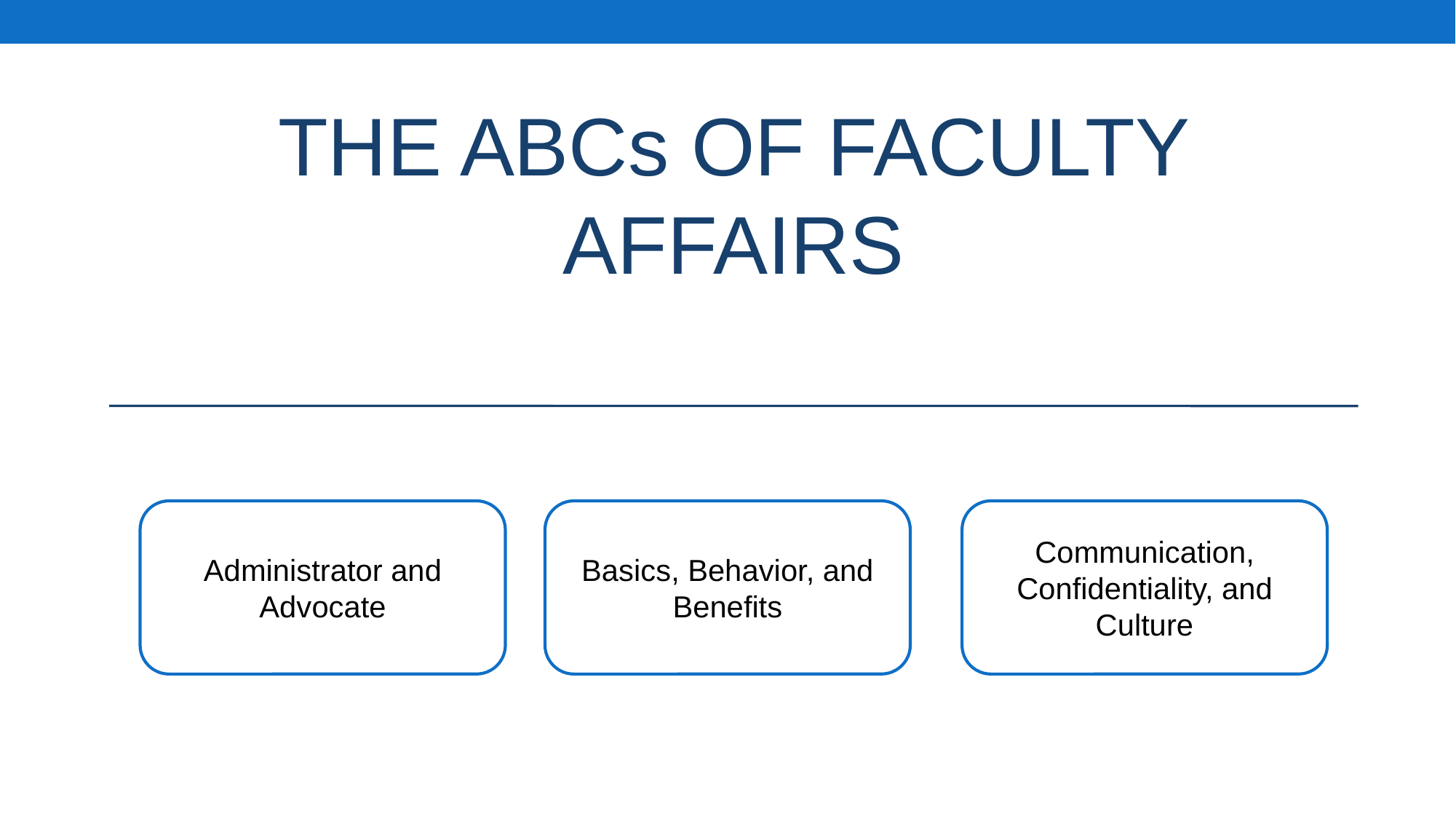

# THE ABCs OF FACULTY AFFAIRS
Basics, Behavior, and Benefits
Communication, Confidentiality, and Culture
Administrator and Advocate

## Slide 11
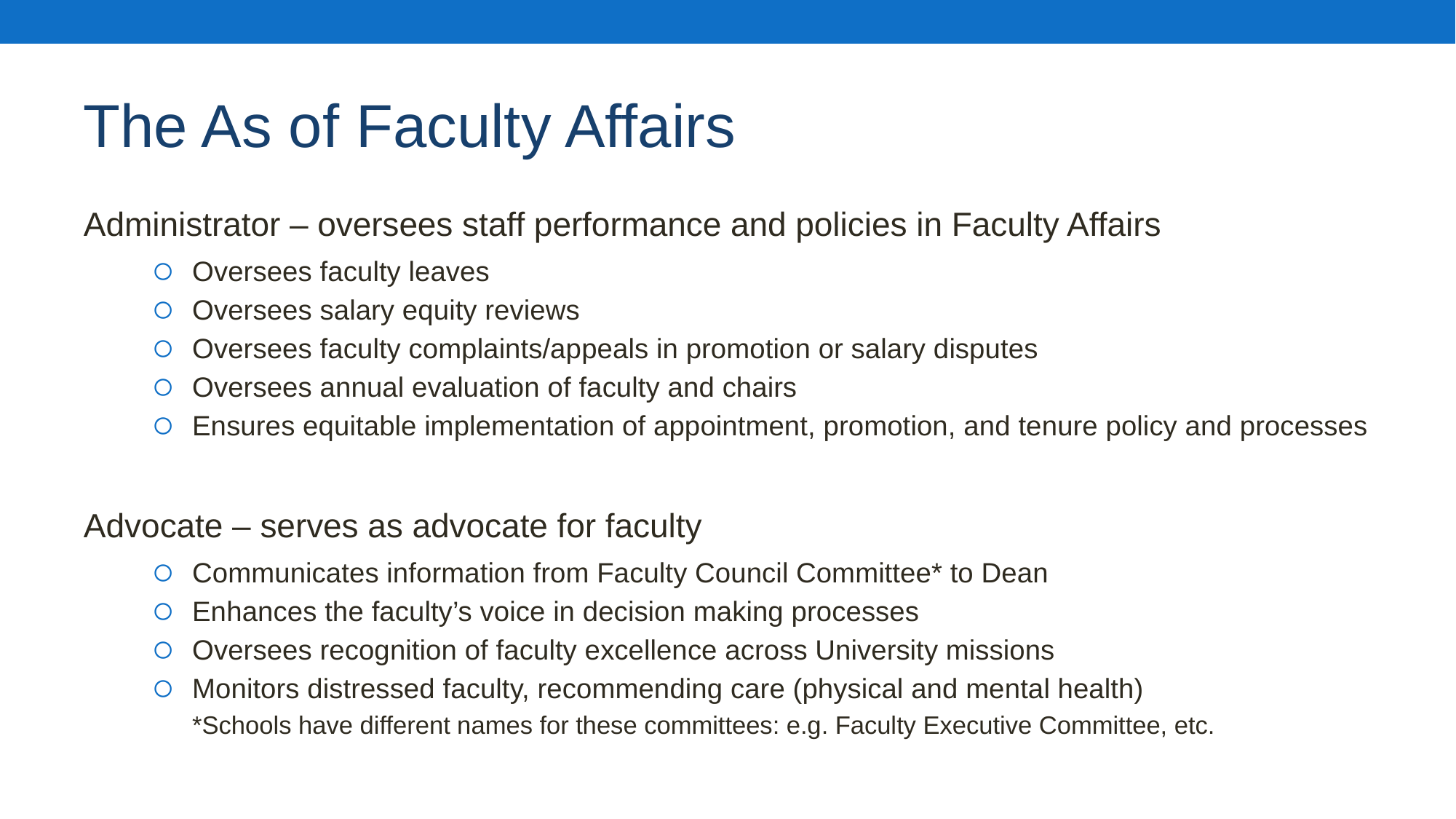

# The As of Faculty Affairs
Administrator – oversees staff performance and policies in Faculty Affairs
Oversees faculty leaves
Oversees salary equity reviews
Oversees faculty complaints/appeals in promotion or salary disputes
Oversees annual evaluation of faculty and chairs
Ensures equitable implementation of appointment, promotion, and tenure policy and processes
Advocate – serves as advocate for faculty
Communicates information from Faculty Council Committee* to Dean
Enhances the faculty’s voice in decision making processes
Oversees recognition of faculty excellence across University missions
Monitors distressed faculty, recommending care (physical and mental health)
*Schools have different names for these committees: e.g. Faculty Executive Committee, etc.

## Slide 12
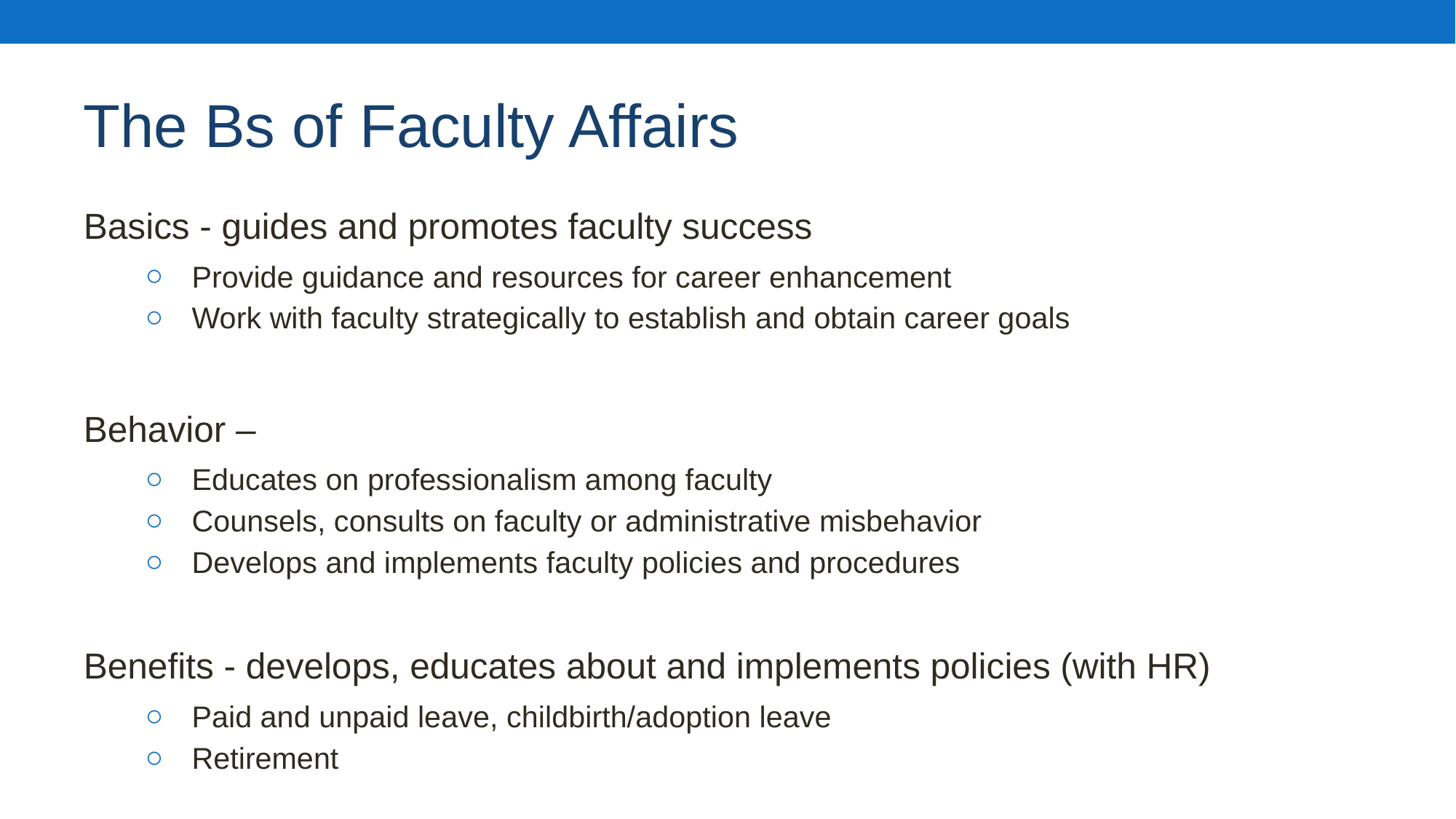

# The Bs of Faculty Affairs
Basics - guides and promotes faculty success
Provide guidance and resources for career enhancement
Work with faculty strategically to establish and obtain career goals
Behavior –
Educates on professionalism among faculty
Counsels, consults on faculty or administrative misbehavior
Develops and implements faculty policies and procedures
Benefits - develops, educates about and implements policies (with HR)
Paid and unpaid leave, childbirth/adoption leave
Retirement

## Slide 13
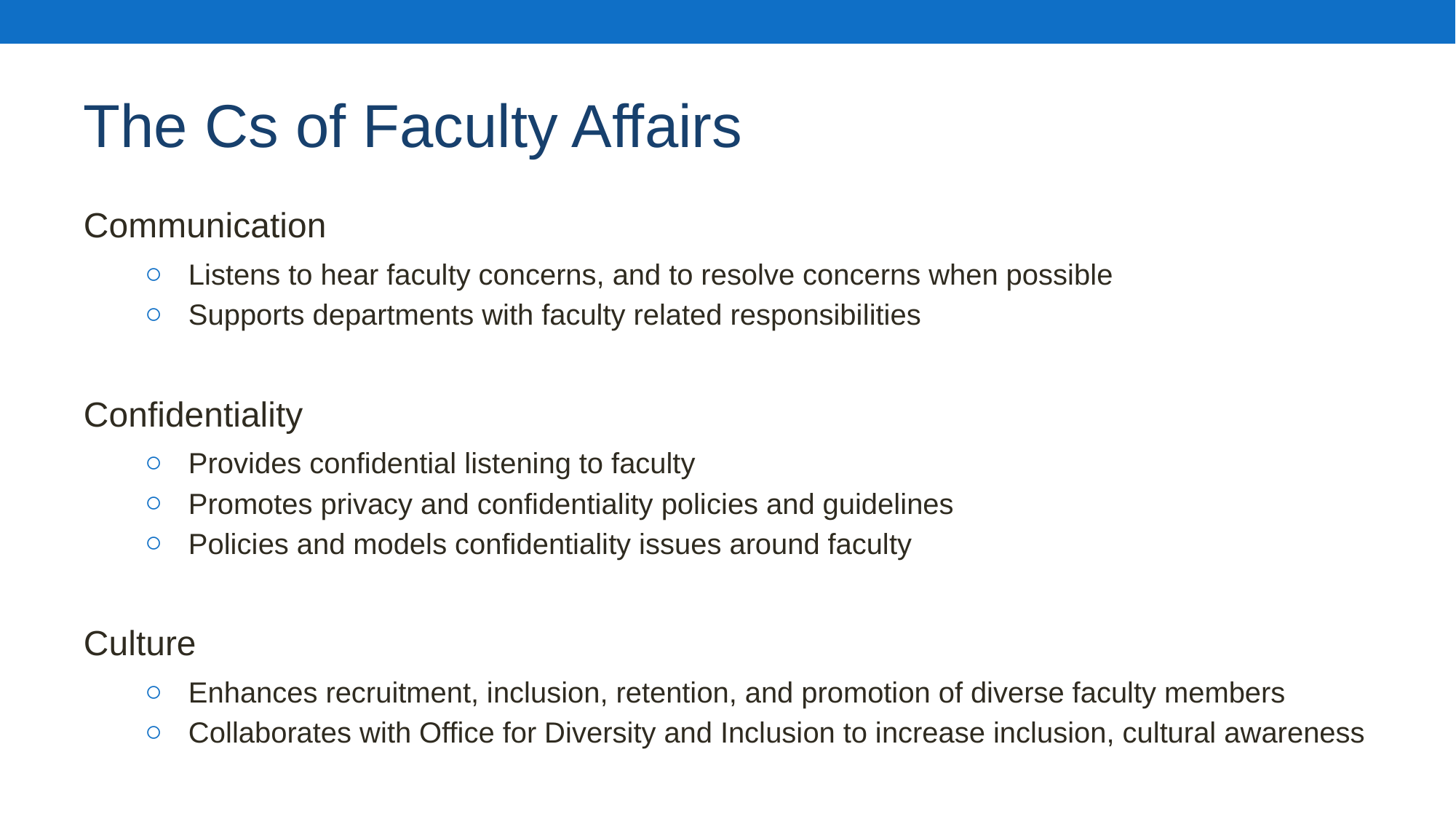

# The Cs of Faculty Affairs
Communication
Listens to hear faculty concerns, and to resolve concerns when possible
Supports departments with faculty related responsibilities
Confidentiality
Provides confidential listening to faculty
Promotes privacy and confidentiality policies and guidelines
Policies and models confidentiality issues around faculty
Culture
Enhances recruitment, inclusion, retention, and promotion of diverse faculty members
Collaborates with Office for Diversity and Inclusion to increase inclusion, cultural awareness

## Slide 14
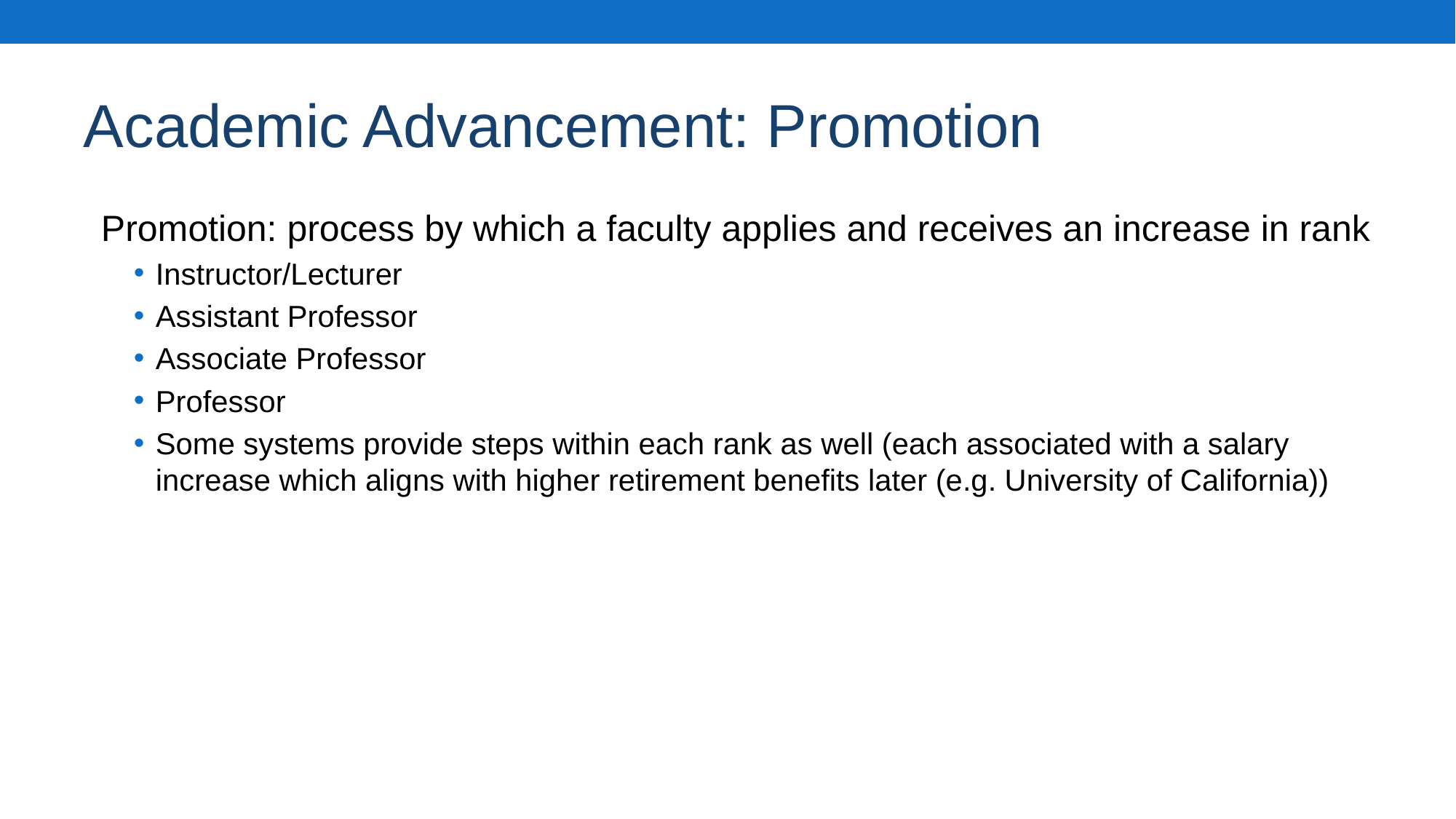

# Academic Advancement: Promotion
Promotion: process by which a faculty applies and receives an increase in rank
Instructor/Lecturer
Assistant Professor
Associate Professor
Professor
Some systems provide steps within each rank as well (each associated with a salary increase which aligns with higher retirement benefits later (e.g. University of California))

## Slide 15
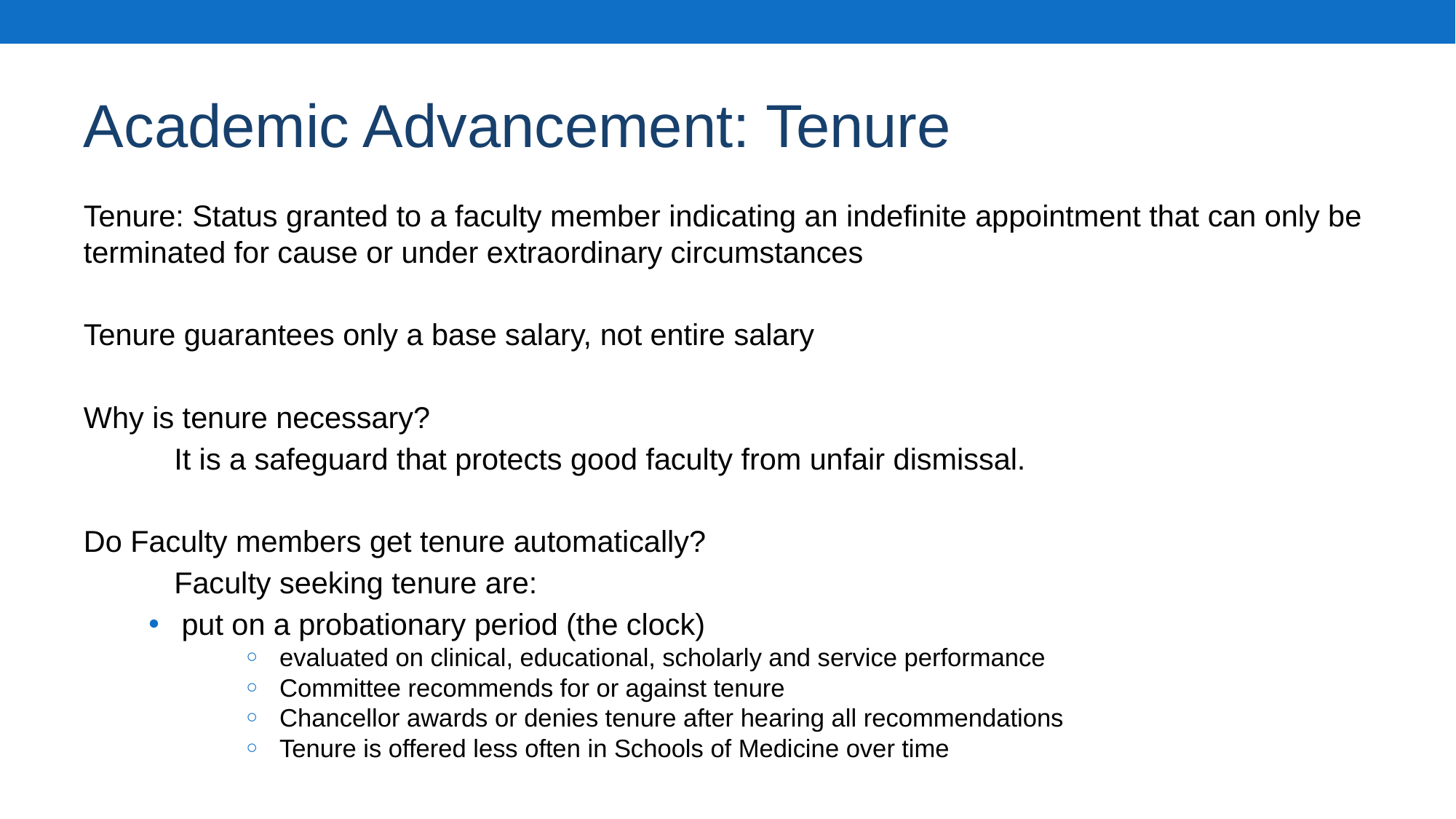

# Academic Advancement: Tenure
Tenure: Status granted to a faculty member indicating an indefinite appointment that can only be terminated for cause or under extraordinary circumstances
Tenure guarantees only a base salary, not entire salary
Why is tenure necessary?
	It is a safeguard that protects good faculty from unfair dismissal.
Do Faculty members get tenure automatically?
	Faculty seeking tenure are:
put on a probationary period (the clock)
evaluated on clinical, educational, scholarly and service performance
Committee recommends for or against tenure
Chancellor awards or denies tenure after hearing all recommendations
Tenure is offered less often in Schools of Medicine over time

## Slide 16
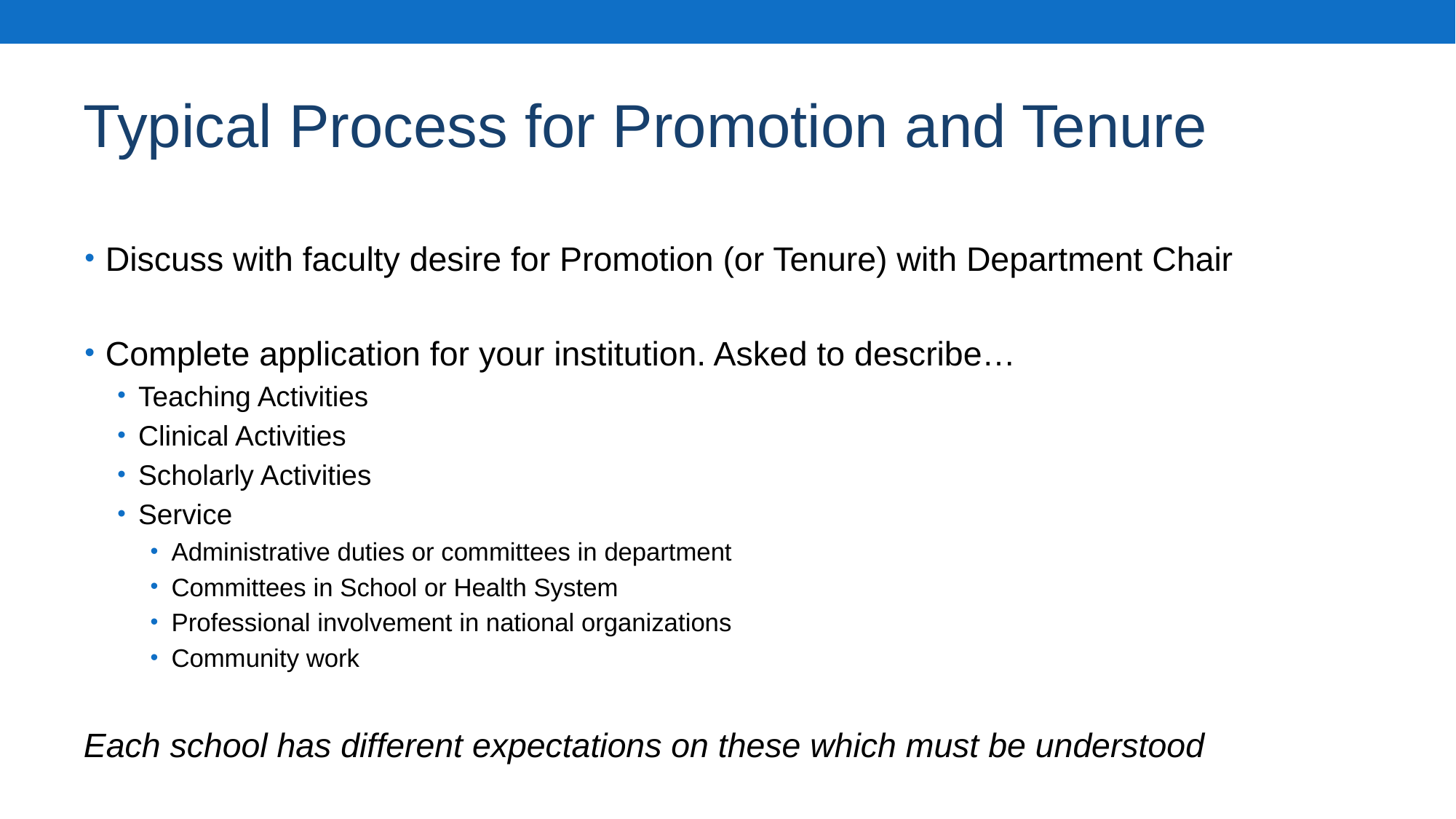

# Typical Process for Promotion and Tenure
Discuss with faculty desire for Promotion (or Tenure) with Department Chair
Complete application for your institution. Asked to describe…
Teaching Activities
Clinical Activities
Scholarly Activities
Service
Administrative duties or committees in department
Committees in School or Health System
Professional involvement in national organizations
Community work
Each school has different expectations on these which must be understood

## Slide 17
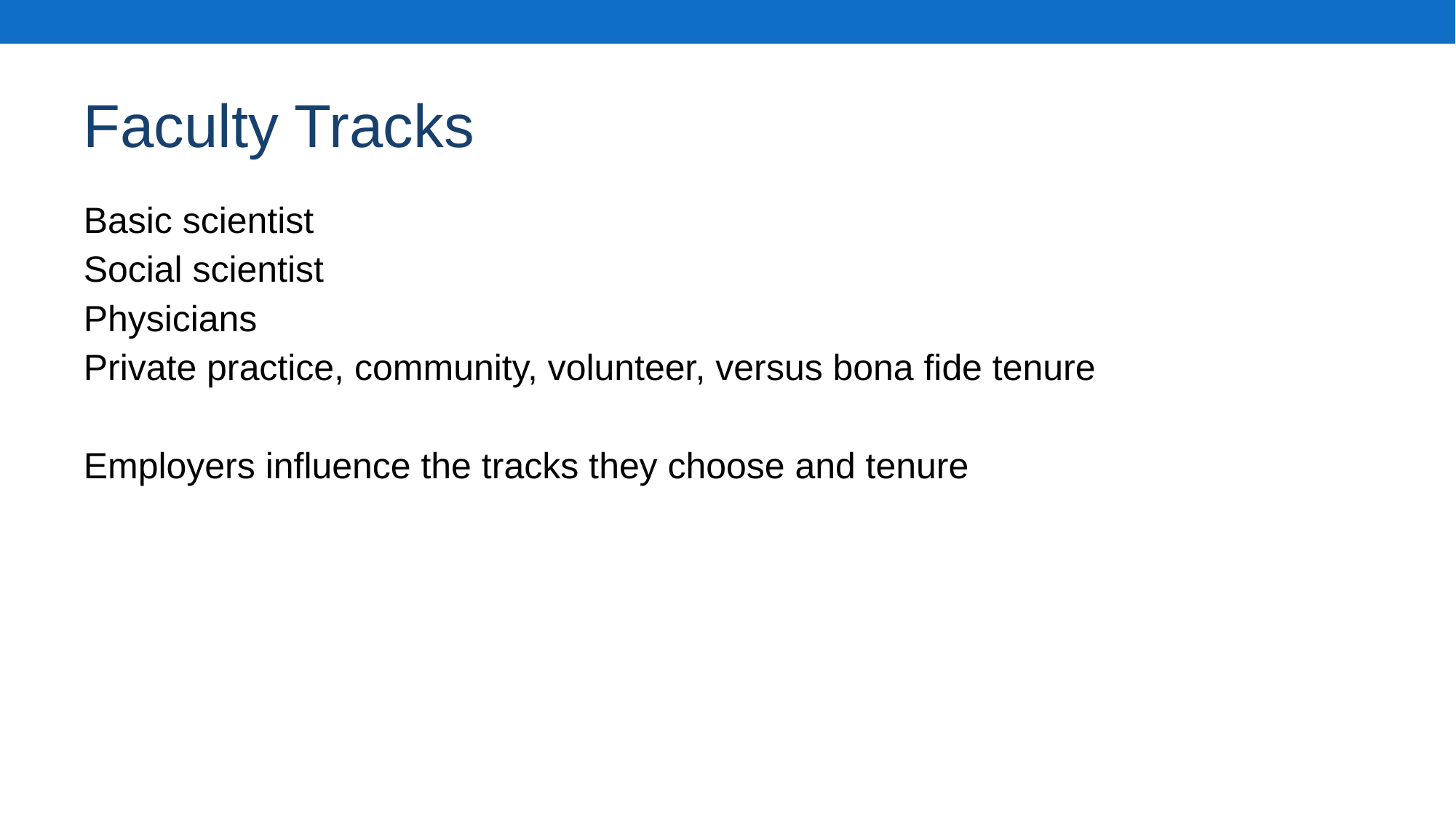

# Faculty Tracks
Basic scientist
Social scientist
Physicians
Private practice, community, volunteer, versus bona fide tenure
Employers influence the tracks they choose and tenure

## Slide 18
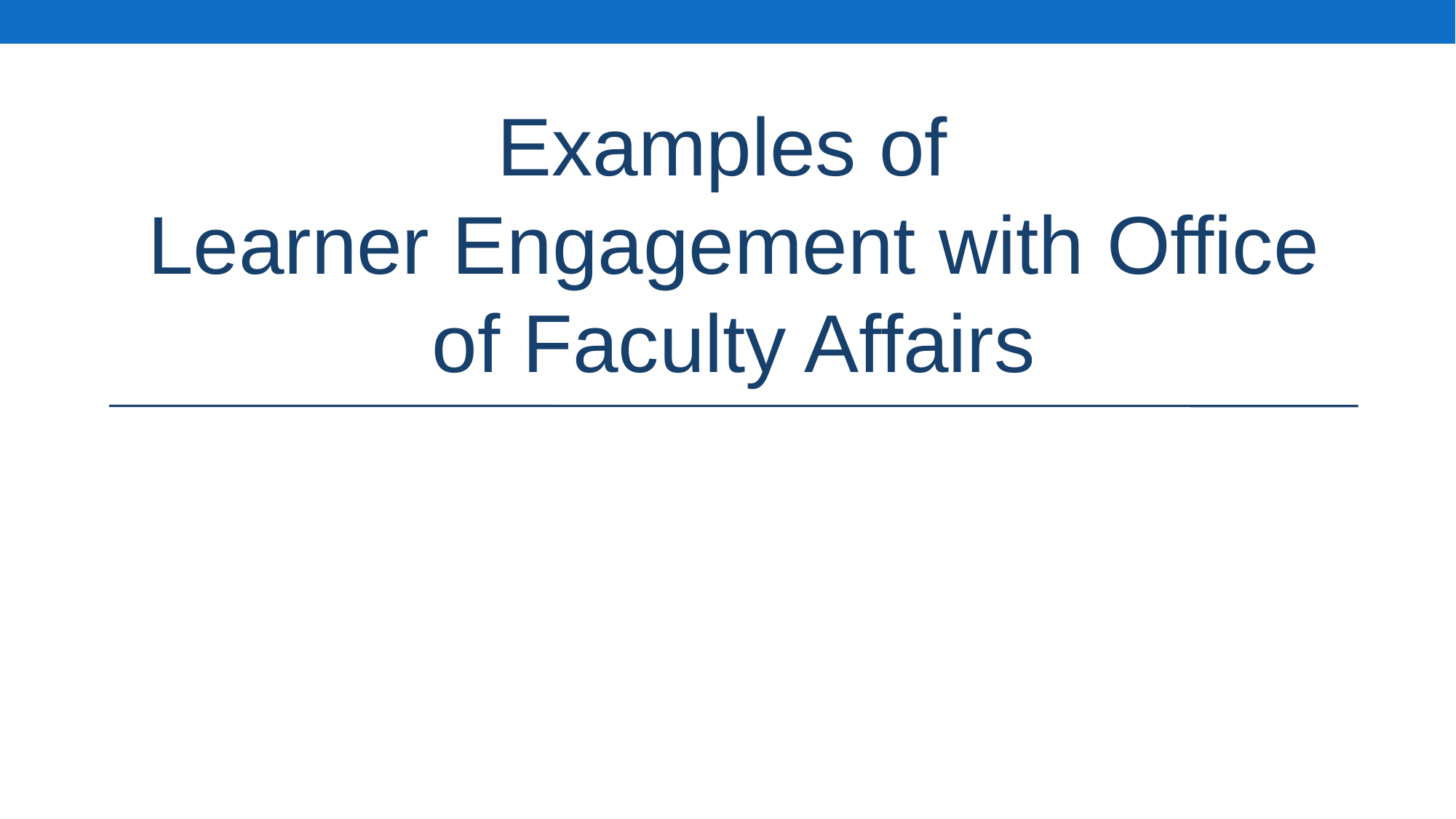

# Examples of
Learner Engagement with Office of Faculty Affairs

## Slide 19
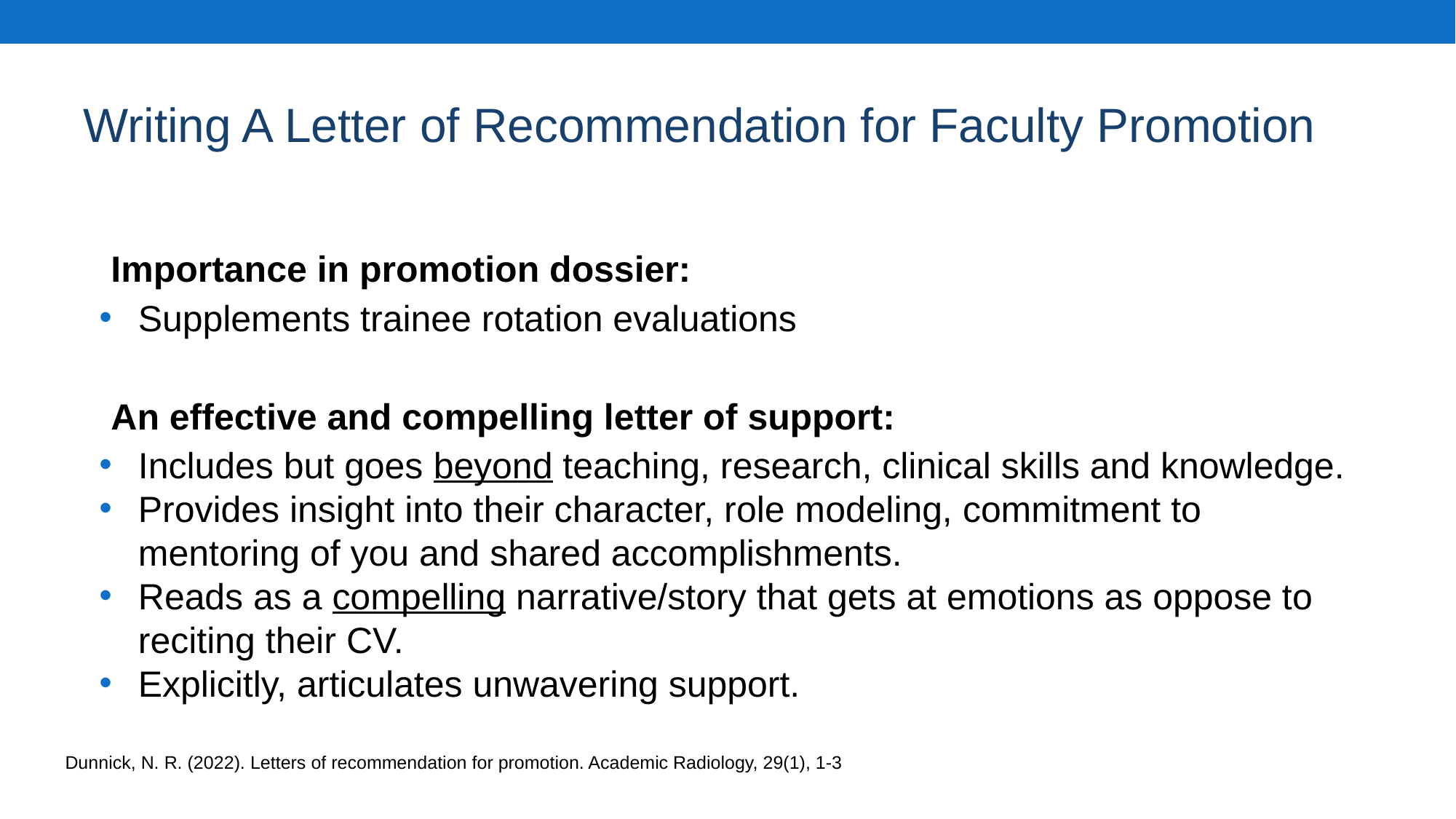

# Writing A Letter of Recommendation for Faculty Promotion
Importance in promotion dossier:
Supplements trainee rotation evaluations
An effective and compelling letter of support:
Includes but goes beyond teaching, research, clinical skills and knowledge.
Provides insight into their character, role modeling, commitment to mentoring of you and shared accomplishments.
Reads as a compelling narrative/story that gets at emotions as oppose to reciting their CV.
Explicitly, articulates unwavering support.
Dunnick, N. R. (2022). Letters of recommendation for promotion. Academic Radiology, 29(1), 1-3

## Slide 20
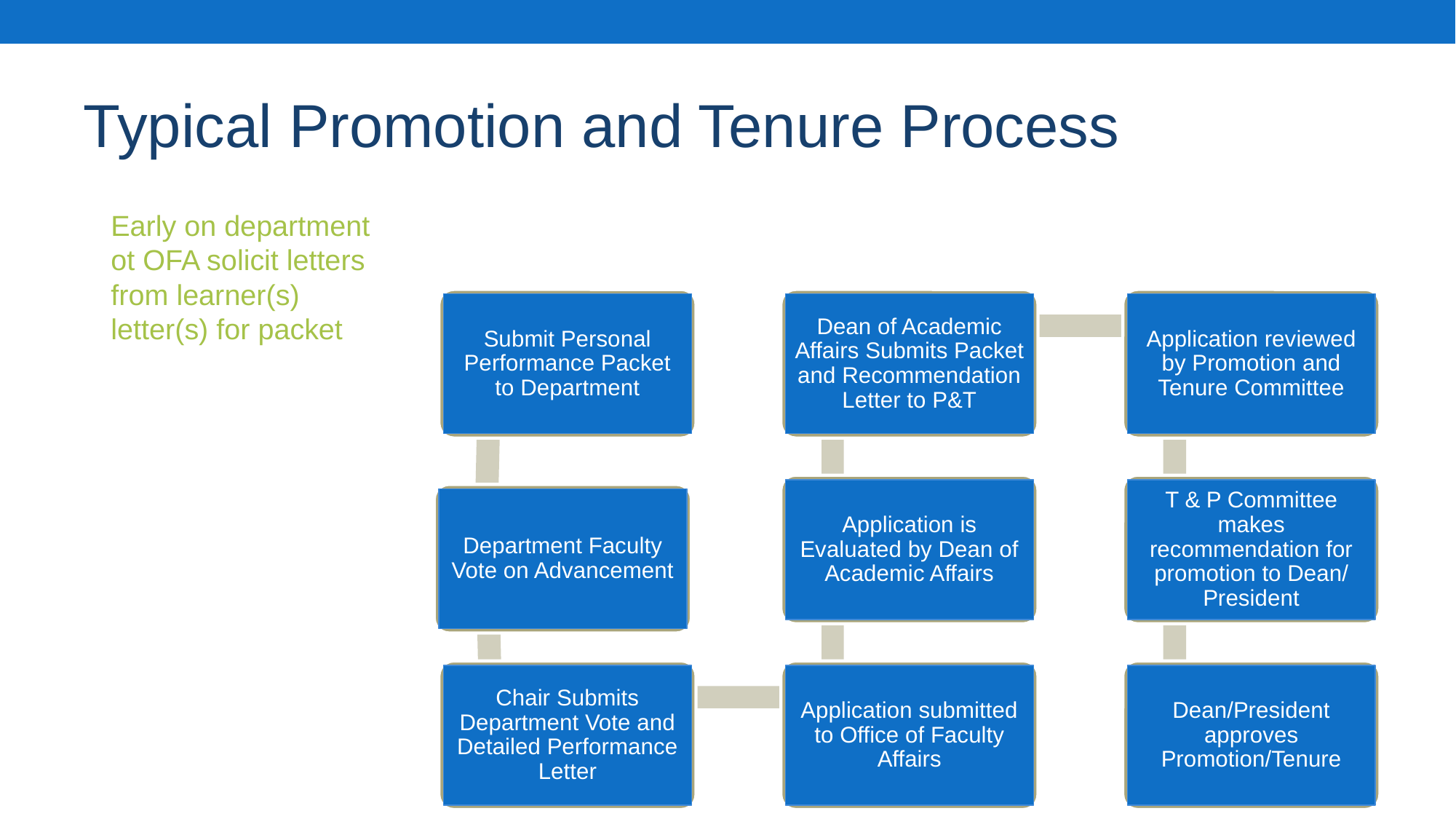

# Typical Promotion and Tenure Process
Early on department ot OFA solicit letters from learner(s) letter(s) for packet
Submit Personal Performance Packet to Department
Dean of Academic Affairs Submits Packet and Recommendation Letter to P&T
Application reviewed by Promotion and Tenure Committee
Application is Evaluated by Dean of Academic Affairs
T & P Committee makes recommendation for promotion to Dean/ President
Department Faculty Vote on Advancement
Chair Submits Department Vote and Detailed Performance Letter
Application submitted to Office of Faculty Affairs
Dean/President approves Promotion/Tenure

## Slide 21
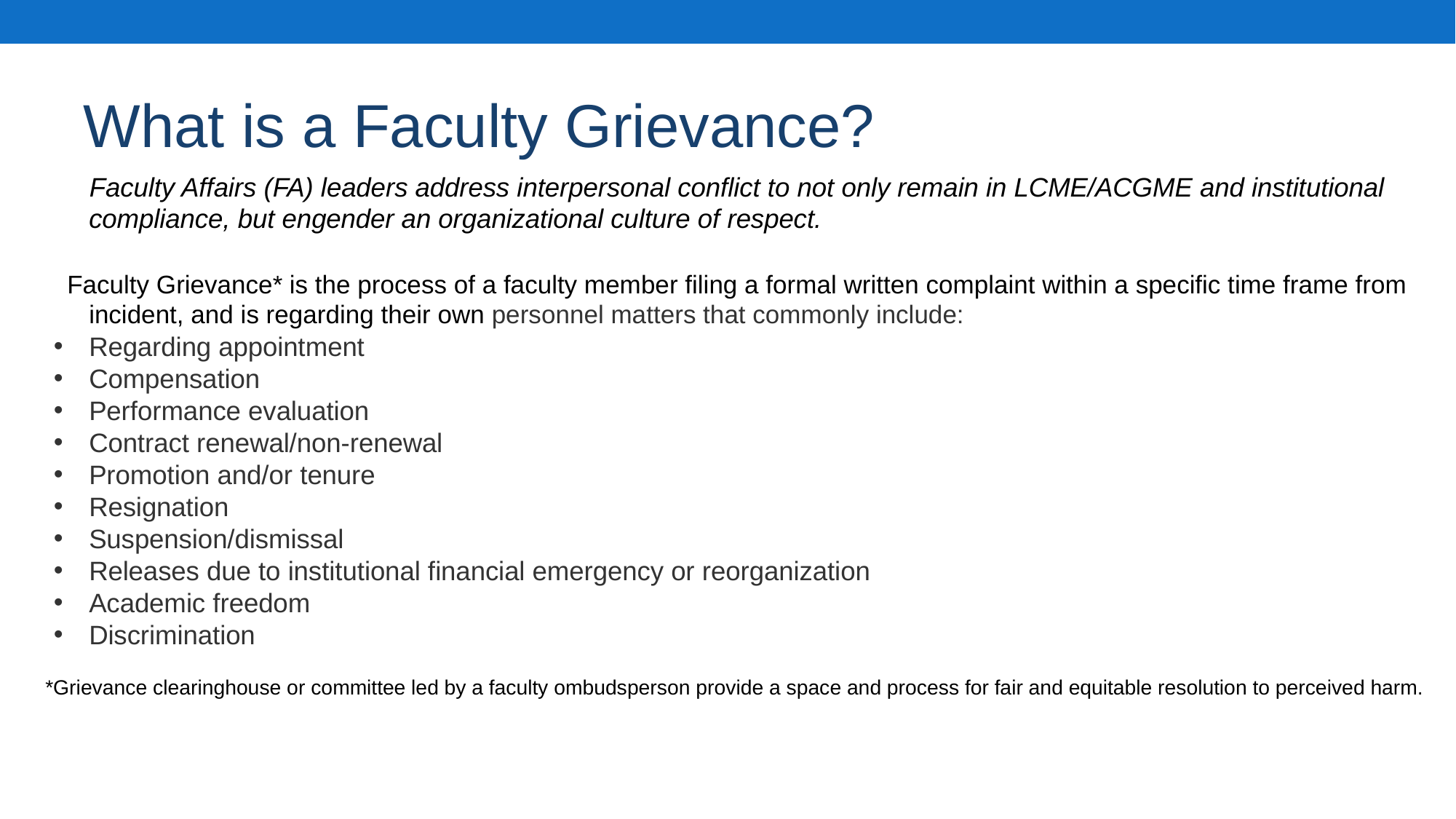

# What is a Faculty Grievance?
 Faculty Affairs (FA) leaders address interpersonal conflict to not only remain in LCME/ACGME and institutional compliance, but engender an organizational culture of respect.
Faculty Grievance* is the process of a faculty member filing a formal written complaint within a specific time frame from incident, and is regarding their own personnel matters that commonly include:
Regarding appointment
Compensation
Performance evaluation
Contract renewal/non-renewal
Promotion and/or tenure
Resignation
Suspension/dismissal
Releases due to institutional financial emergency or reorganization
Academic freedom
Discrimination
*Grievance clearinghouse or committee led by a faculty ombudsperson provide a space and process for fair and equitable resolution to perceived harm.

## Slide 22
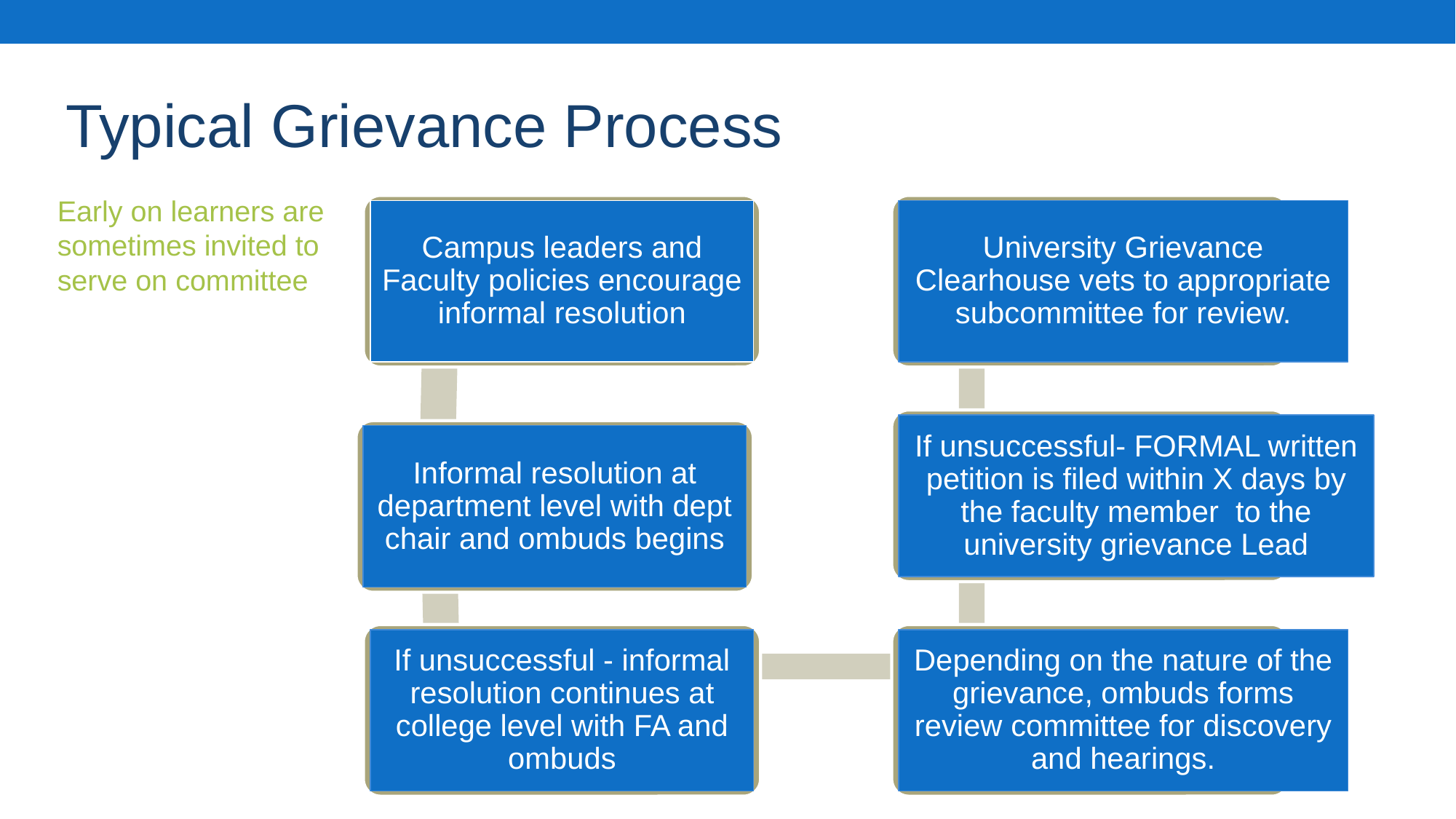

# Typical Grievance Process
Early on learners are sometimes invited to serve on committee
University Grievance Clearhouse vets to appropriate subcommittee for review.
Campus leaders and Faculty policies encourage informal resolution
If unsuccessful- FORMAL written petition is filed within X days by the faculty member to the university grievance Lead
Informal resolution at department level with dept chair and ombuds begins
If unsuccessful - informal resolution continues at college level with FA and ombuds
Depending on the nature of the grievance, ombuds forms review committee for discovery and hearings.

## Slide 23
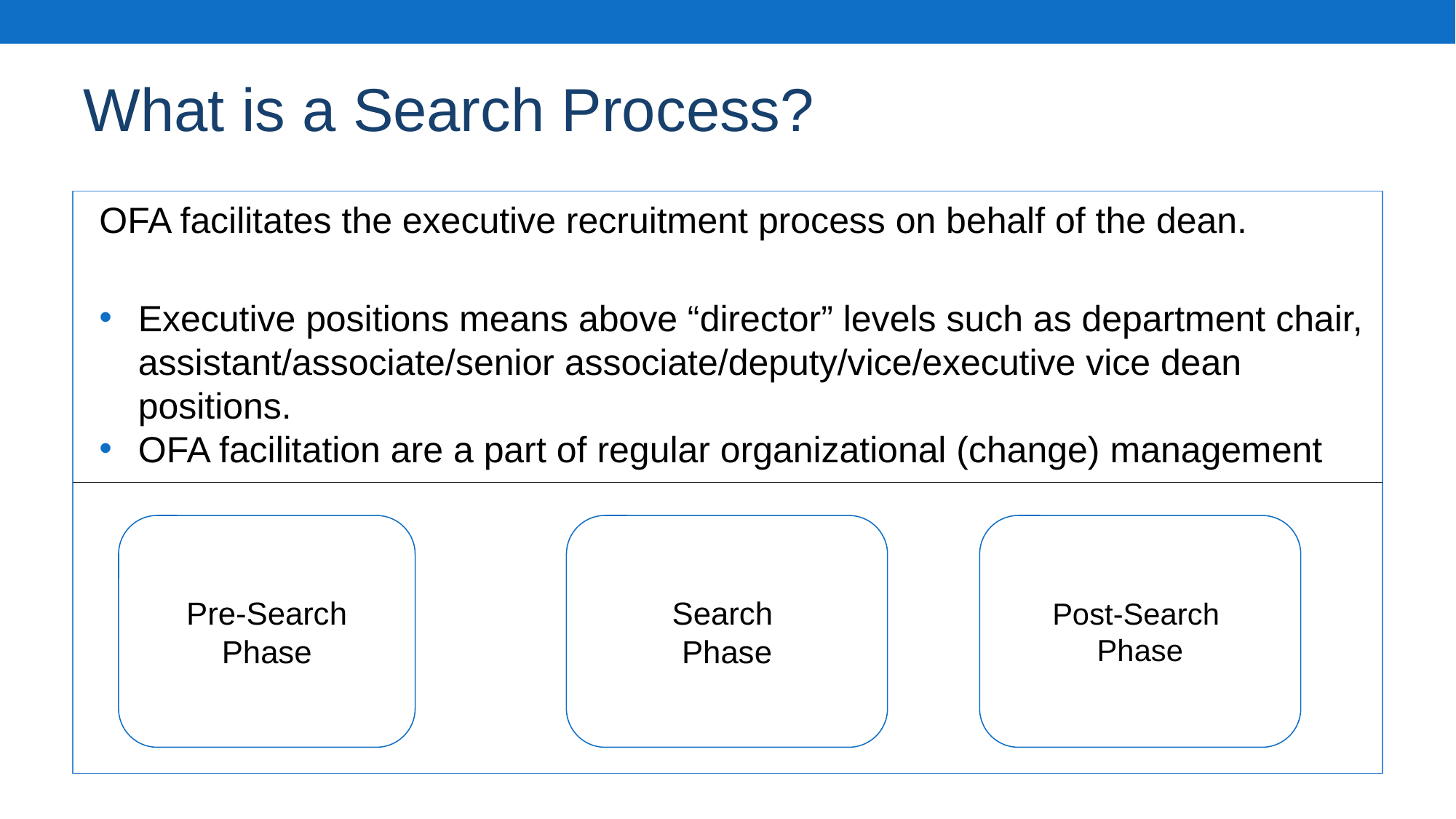

# What is a Search Process?
OFA facilitates the executive recruitment process on behalf of the dean.
Executive positions means above “director” levels such as department chair, assistant/associate/senior associate/deputy/vice/executive vice dean positions.
OFA facilitation are a part of regular organizational (change) management
Search
Phase
Pre-Search Phase
Post-Search
Phase

## Slide 24
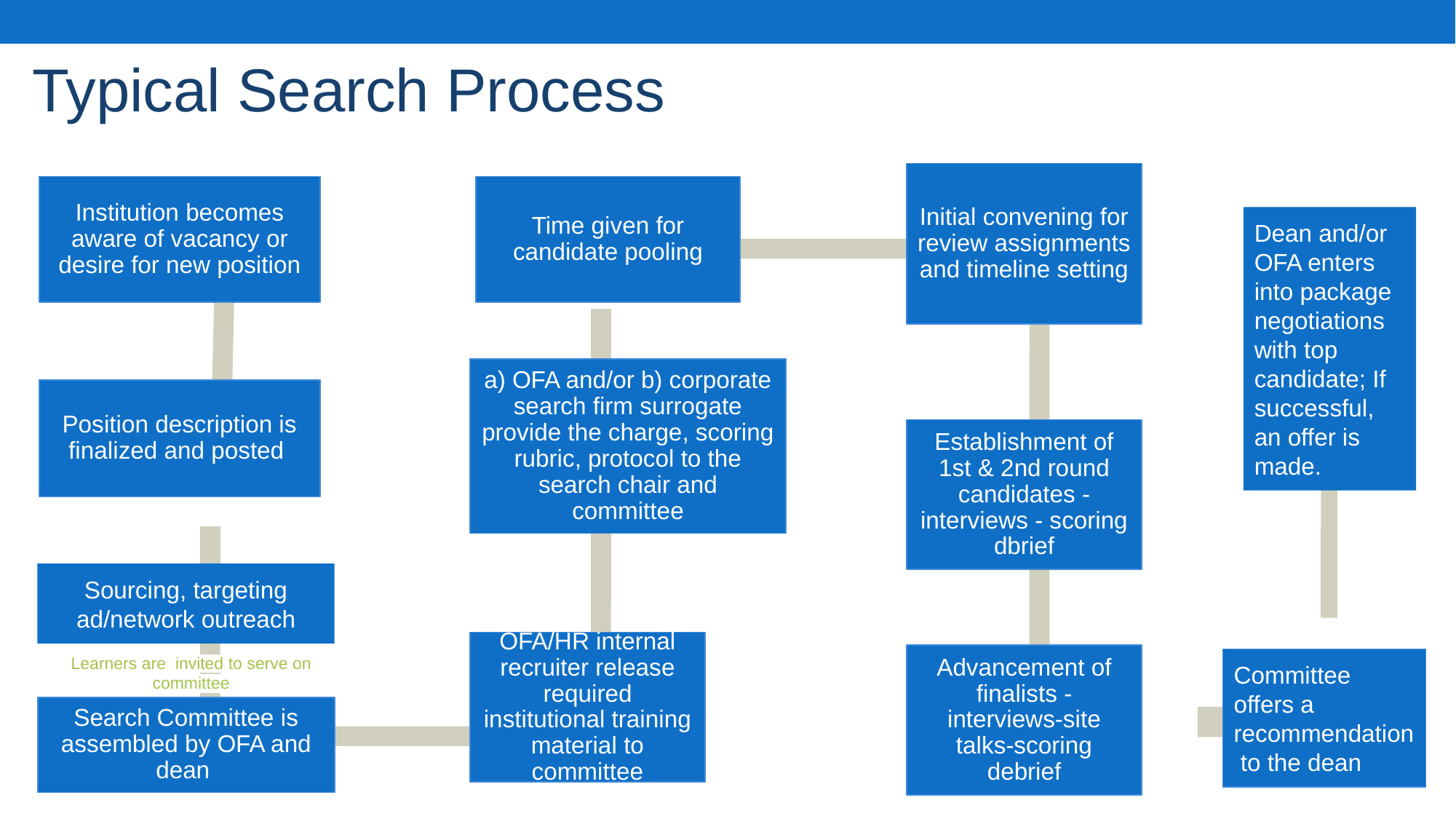

# Typical Search Process
Initial convening for review assignments and timeline setting
Institution becomes aware of vacancy or desire for new position
Time given for candidate pooling
Dean and/or OFA enters into package negotiations with top candidate; If successful, an offer is made.
a) OFA and/or b) corporate search firm surrogate provide the charge, scoring rubric, protocol to the search chair and committee
Position description is finalized and posted
Establishment of 1st & 2nd round candidates - interviews - scoring dbrief
Sourcing, targeting ad/network outreach
OFA/HR internal recruiter release required institutional training material to committee
Learners are invited to serve on committee
Advancement of finalists - interviews-site talks-scoring debrief
Committee offers a recommendation to the dean
Search Committee is assembled by OFA and dean

## Slide 25
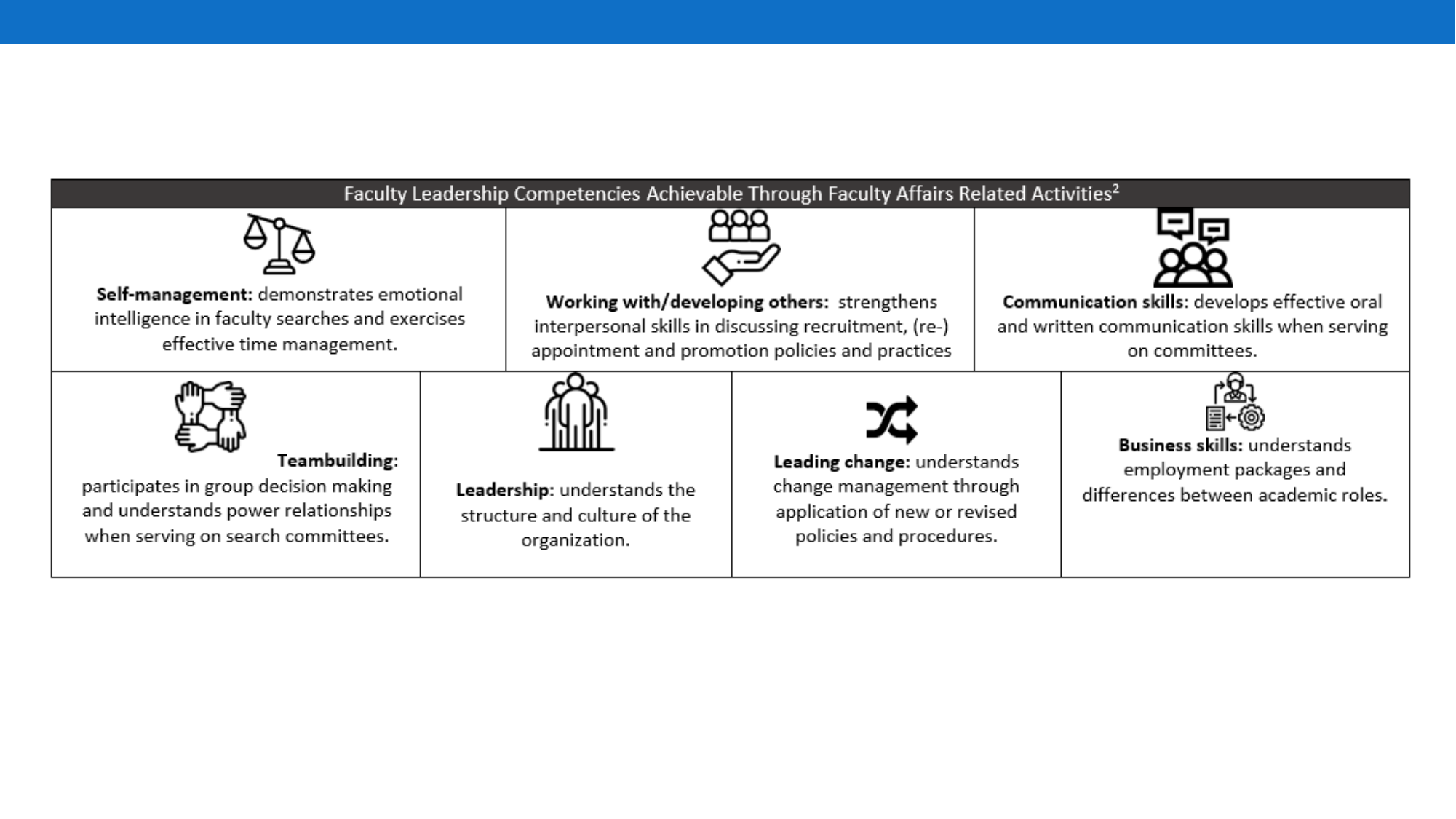

## Slide 26
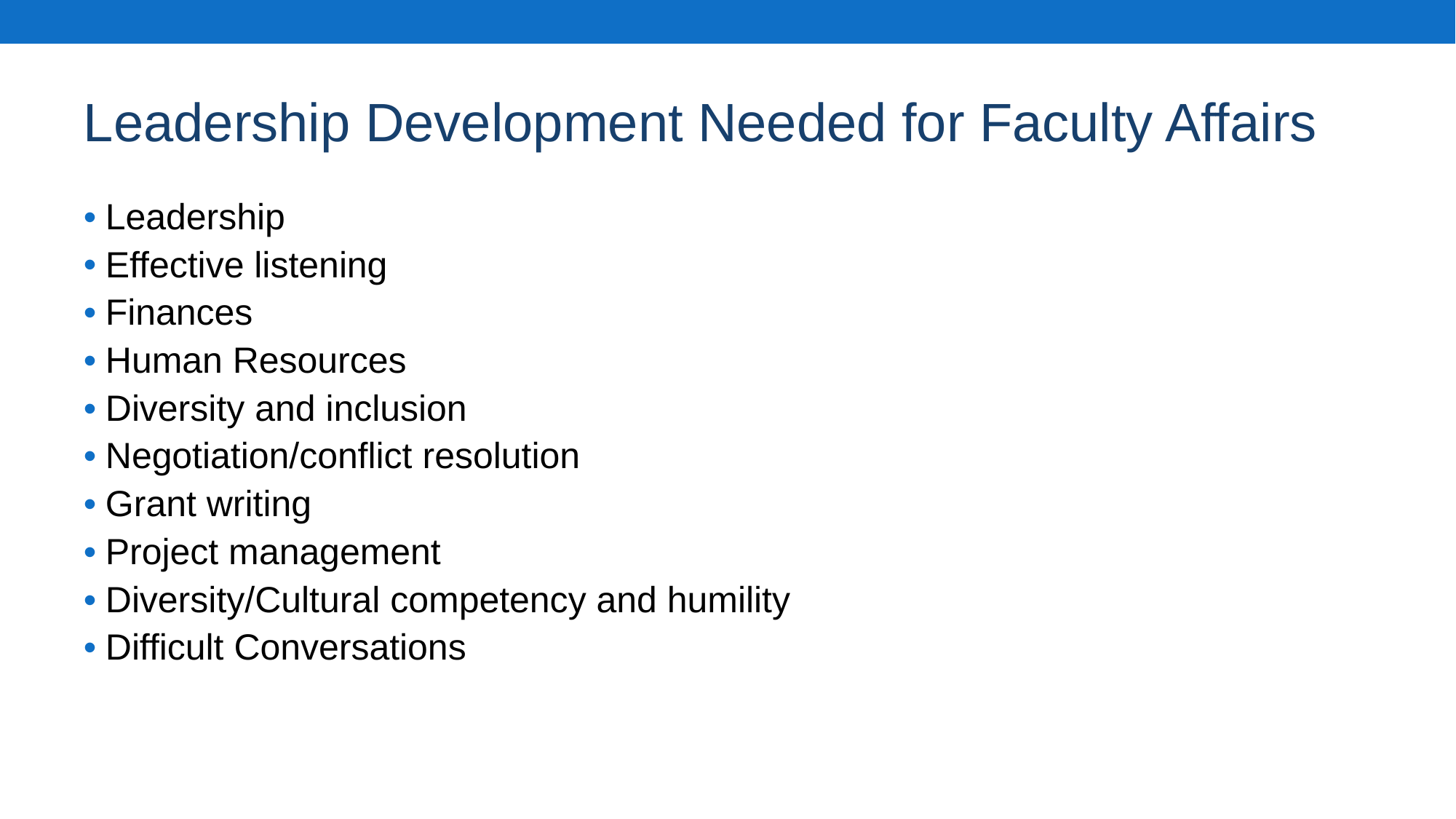

# Leadership Development Needed for Faculty Affairs
Leadership
Effective listening
Finances
Human Resources
Diversity and inclusion
Negotiation/conflict resolution
Grant writing
Project management
Diversity/Cultural competency and humility
Difficult Conversations

## Slide 27
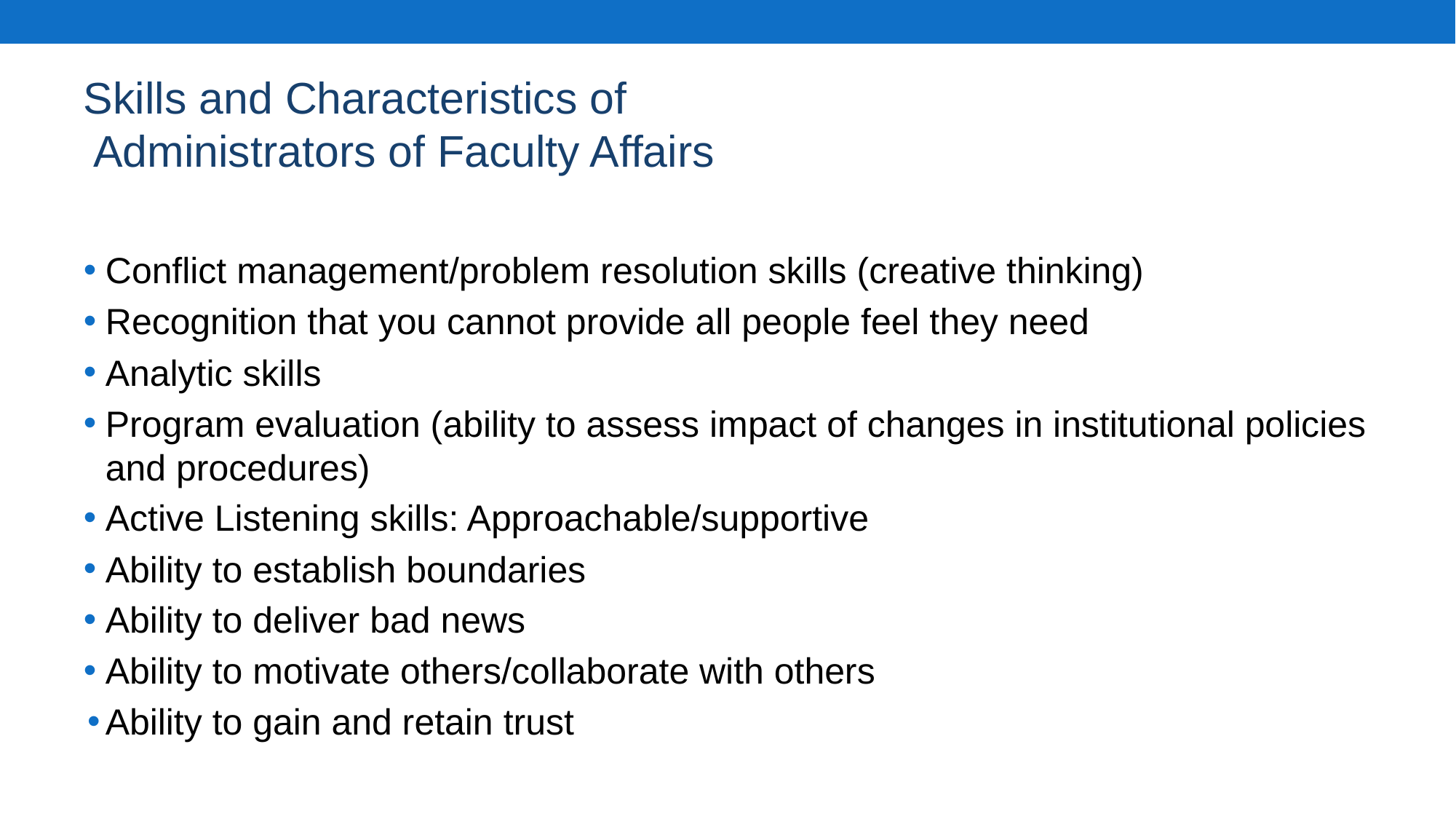

# Skills and Characteristics of Administrators of Faculty Affairs
Conflict management/problem resolution skills (creative thinking)
Recognition that you cannot provide all people feel they need
Analytic skills
Program evaluation (ability to assess impact of changes in institutional policies and procedures)
Active Listening skills: Approachable/supportive
Ability to establish boundaries
Ability to deliver bad news
Ability to motivate others/collaborate with others
Ability to gain and retain trust

## Slide 28
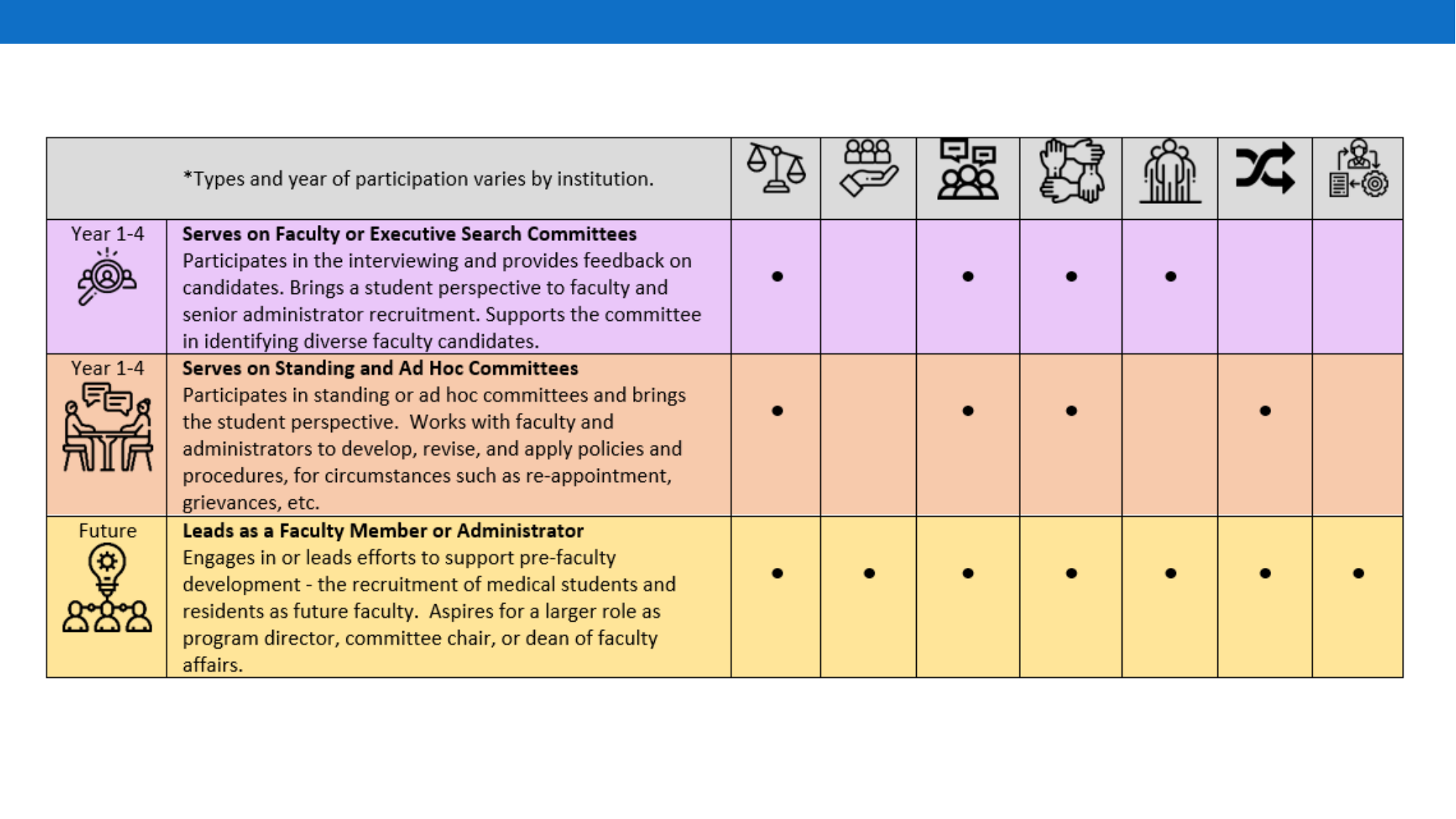

## Slide 29
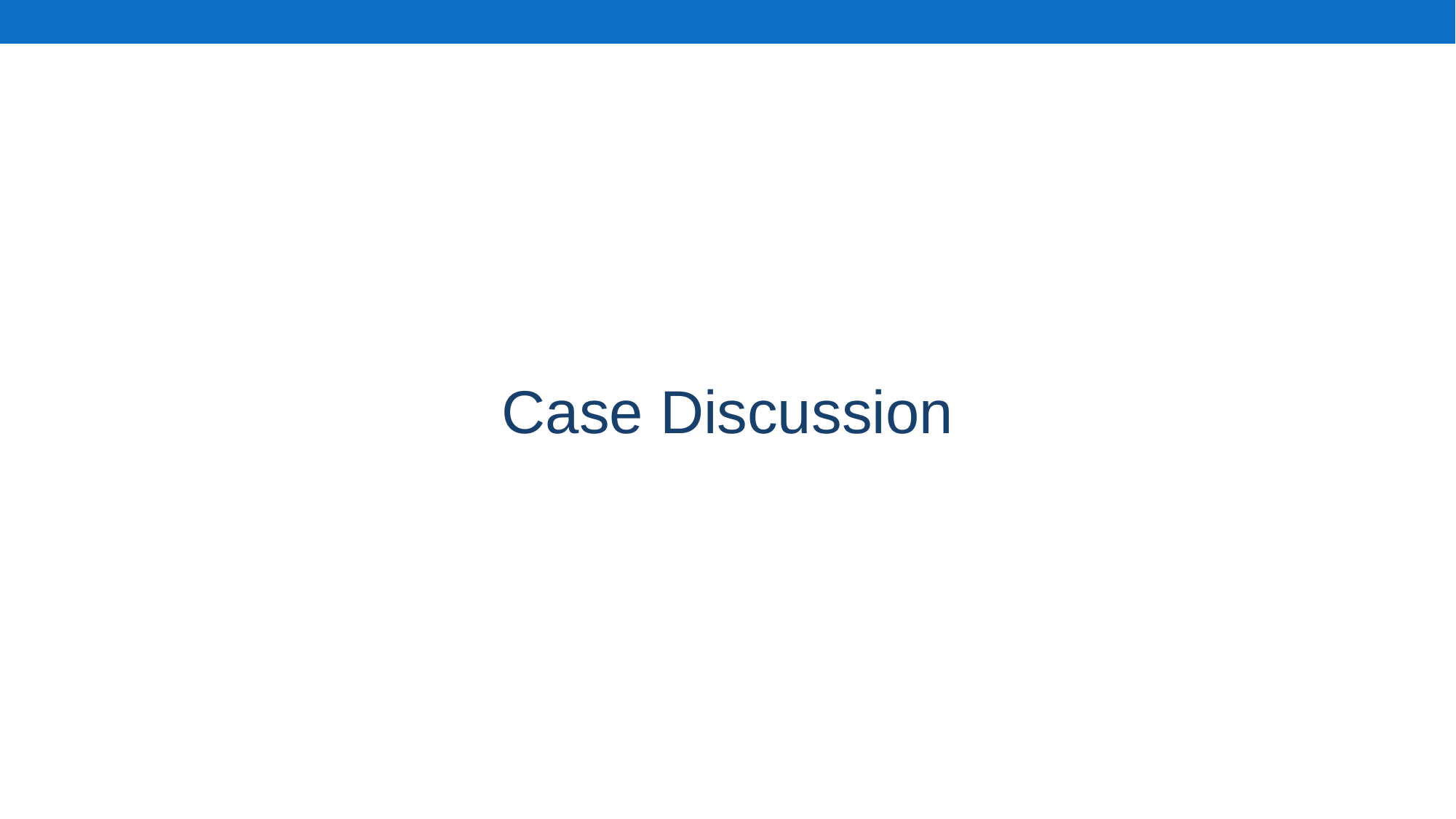

# Case Discussion

## Slide 30
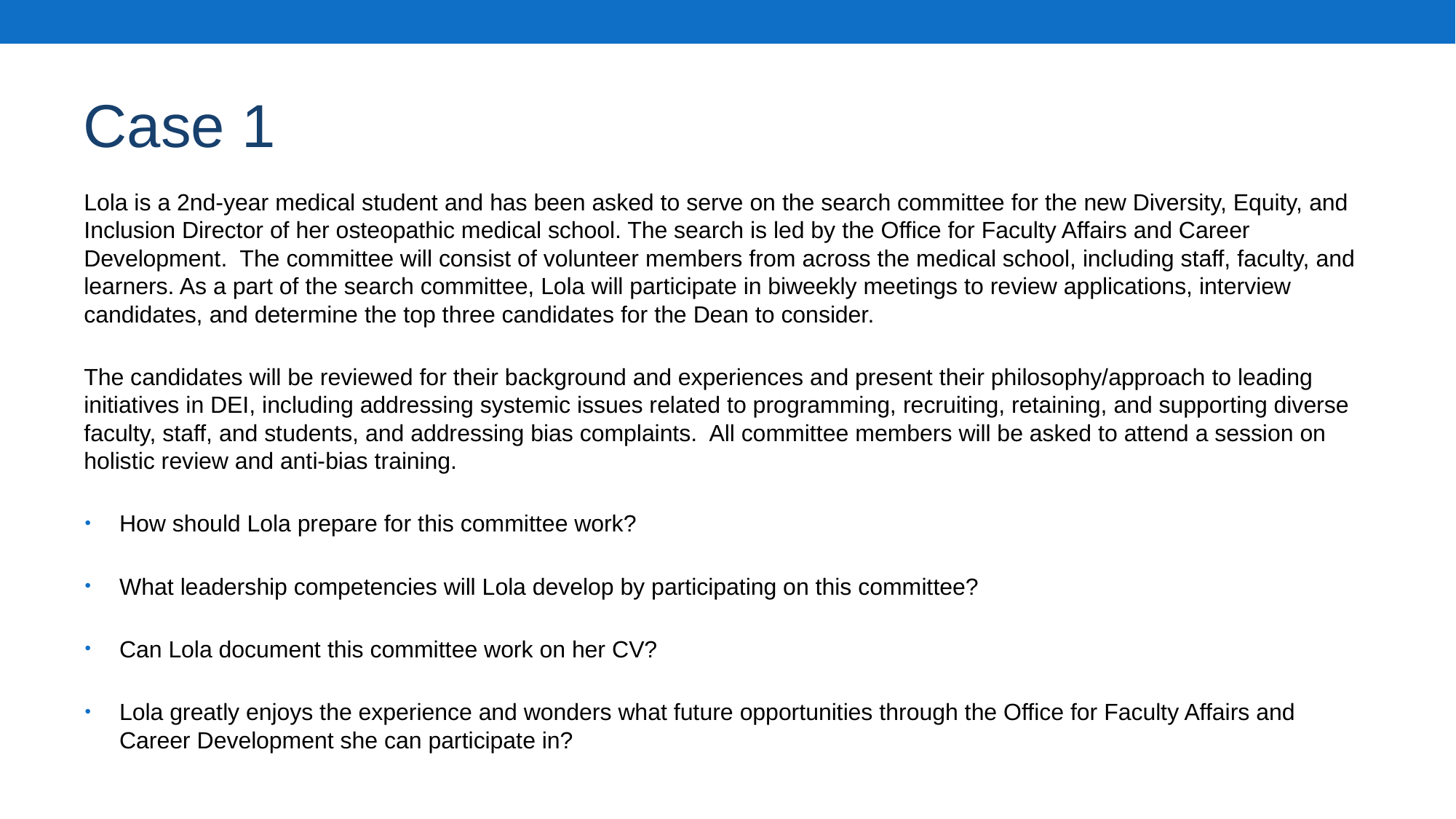

# Case 1
Lola is a 2nd-year medical student and has been asked to serve on the search committee for the new Diversity, Equity, and Inclusion Director of her osteopathic medical school. The search is led by the Office for Faculty Affairs and Career Development. The committee will consist of volunteer members from across the medical school, including staff, faculty, and learners. As a part of the search committee, Lola will participate in biweekly meetings to review applications, interview candidates, and determine the top three candidates for the Dean to consider.
The candidates will be reviewed for their background and experiences and present their philosophy/approach to leading initiatives in DEI, including addressing systemic issues related to programming, recruiting, retaining, and supporting diverse faculty, staff, and students, and addressing bias complaints. All committee members will be asked to attend a session on holistic review and anti-bias training.
How should Lola prepare for this committee work?
What leadership competencies will Lola develop by participating on this committee?
Can Lola document this committee work on her CV?
Lola greatly enjoys the experience and wonders what future opportunities through the Office for Faculty Affairs and Career Development she can participate in?

## Slide 31
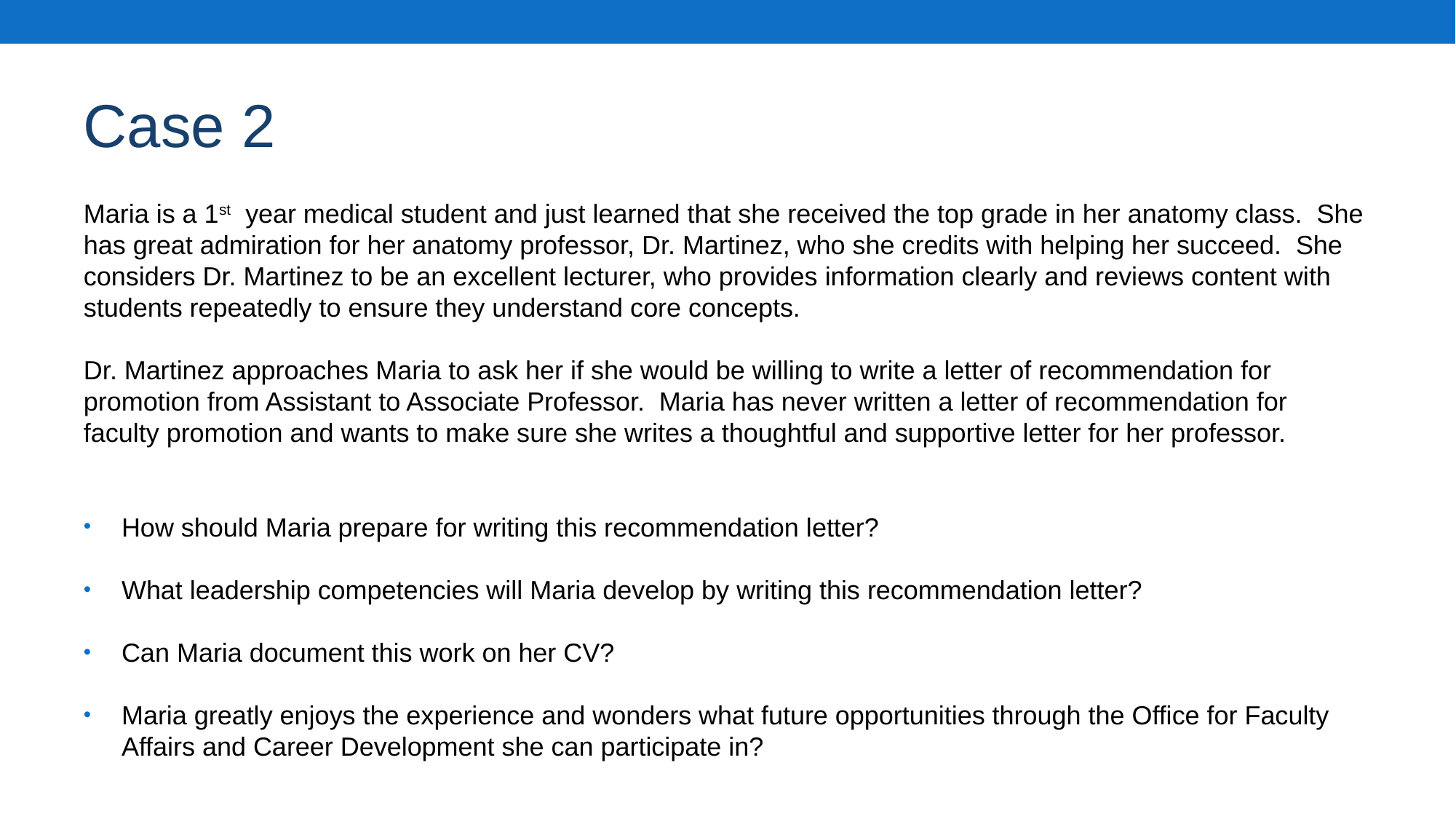

# Case 2
Maria is a 1st year medical student and just learned that she received the top grade in her anatomy class. She has great admiration for her anatomy professor, Dr. Martinez, who she credits with helping her succeed. She considers Dr. Martinez to be an excellent lecturer, who provides information clearly and reviews content with students repeatedly to ensure they understand core concepts.
Dr. Martinez approaches Maria to ask her if she would be willing to write a letter of recommendation for promotion from Assistant to Associate Professor. Maria has never written a letter of recommendation for faculty promotion and wants to make sure she writes a thoughtful and supportive letter for her professor.
How should Maria prepare for writing this recommendation letter?
What leadership competencies will Maria develop by writing this recommendation letter?
Can Maria document this work on her CV?
Maria greatly enjoys the experience and wonders what future opportunities through the Office for Faculty Affairs and Career Development she can participate in?

## Slide 32
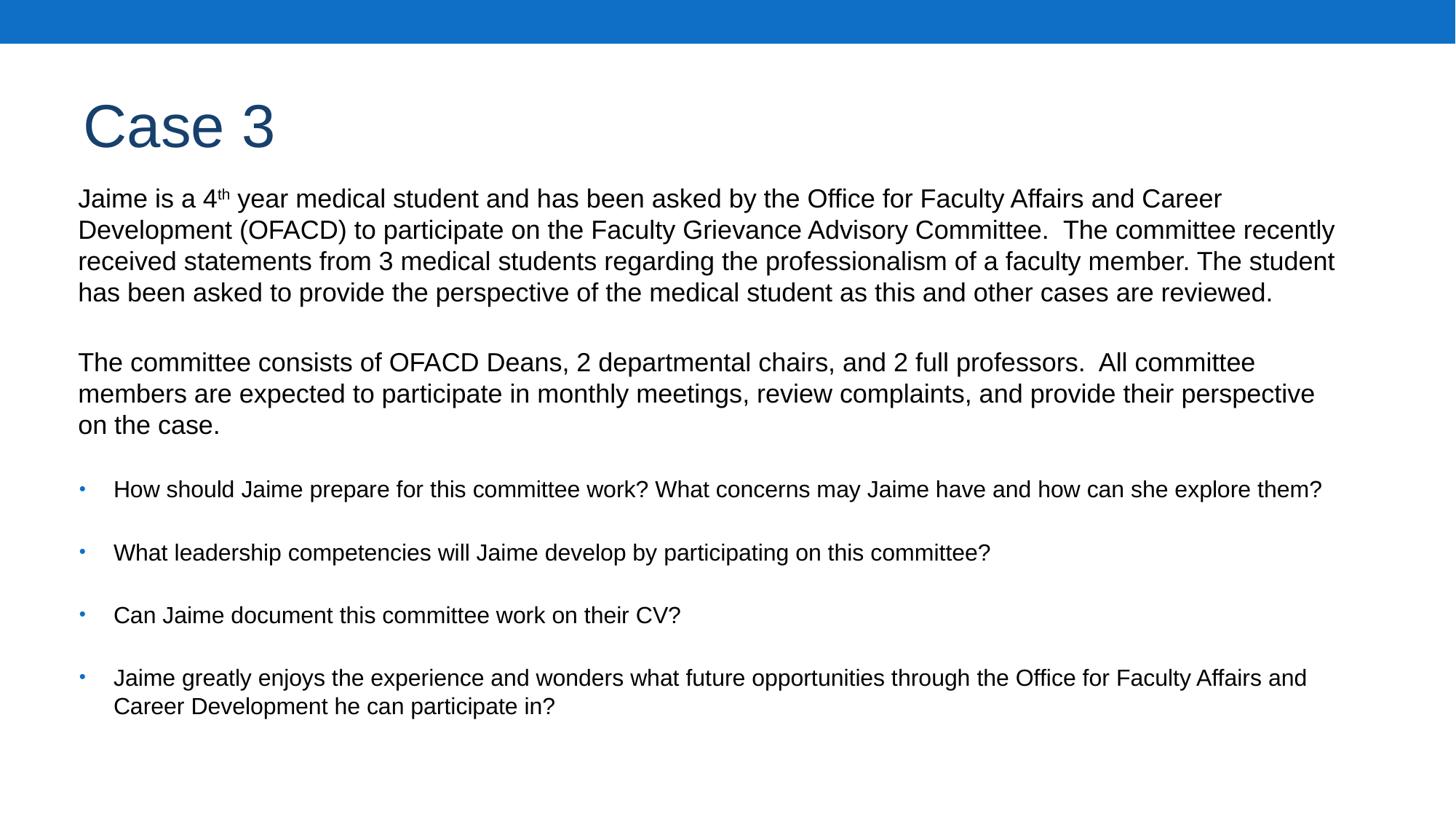

# Case 3
Jaime is a 4th year medical student and has been asked by the Office for Faculty Affairs and Career Development (OFACD) to participate on the Faculty Grievance Advisory Committee. The committee recently received statements from 3 medical students regarding the professionalism of a faculty member. The student has been asked to provide the perspective of the medical student as this and other cases are reviewed.
The committee consists of OFACD Deans, 2 departmental chairs, and 2 full professors. All committee members are expected to participate in monthly meetings, review complaints, and provide their perspective on the case.
How should Jaime prepare for this committee work? What concerns may Jaime have and how can she explore them?
What leadership competencies will Jaime develop by participating on this committee?
Can Jaime document this committee work on their CV?
Jaime greatly enjoys the experience and wonders what future opportunities through the Office for Faculty Affairs and Career Development he can participate in?

## Slide 33
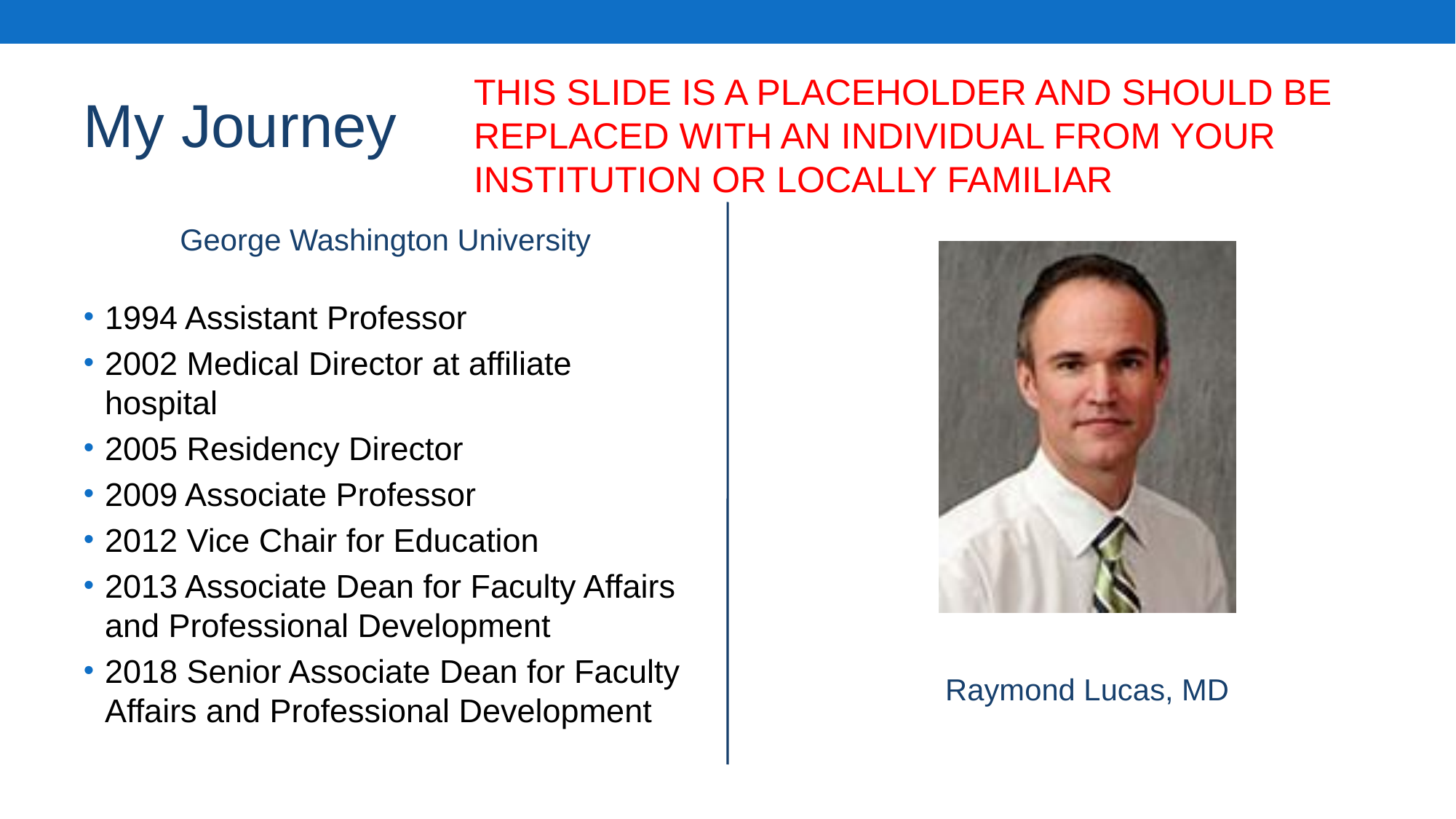

# My Journey
THIS SLIDE IS A PLACEHOLDER AND SHOULD BE REPLACED WITH AN INDIVIDUAL FROM YOUR INSTITUTION OR LOCALLY FAMILIAR
George Washington University
1994 Assistant Professor
2002 Medical Director at affiliate hospital
2005 Residency Director
2009 Associate Professor
2012 Vice Chair for Education
2013 Associate Dean for Faculty Affairs and Professional Development
2018 Senior Associate Dean for Faculty Affairs and Professional Development
Raymond Lucas, MD

## Slide 34
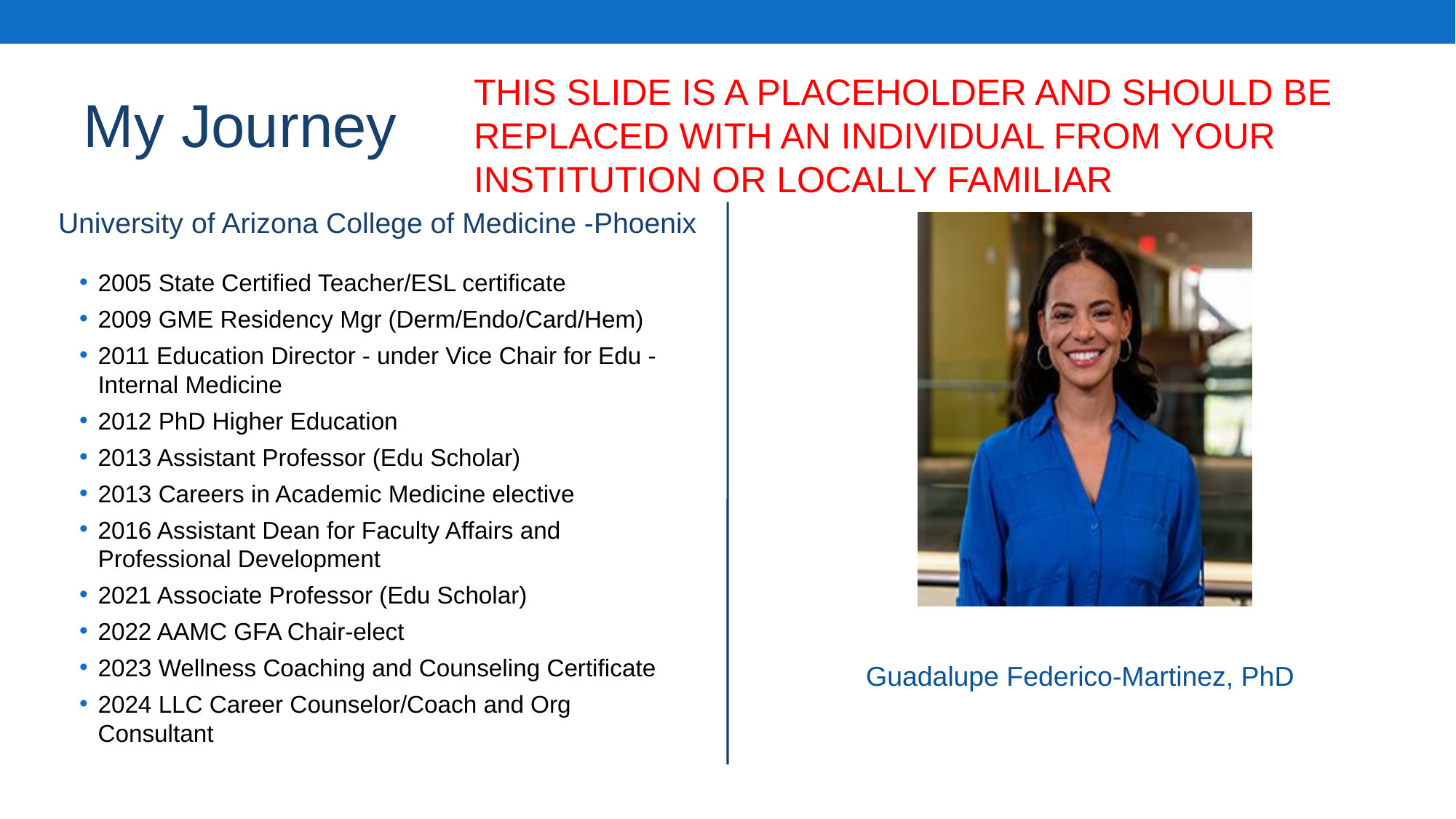

# My Journey
THIS SLIDE IS A PLACEHOLDER AND SHOULD BE REPLACED WITH AN INDIVIDUAL FROM YOUR INSTITUTION OR LOCALLY FAMILIAR
University of Arizona College of Medicine -Phoenix
2005 State Certified Teacher/ESL certificate
2009 GME Residency Mgr (Derm/Endo/Card/Hem)
2011 Education Director - under Vice Chair for Edu - Internal Medicine
2012 PhD Higher Education
2013 Assistant Professor (Edu Scholar)
2013 Careers in Academic Medicine elective
2016 Assistant Dean for Faculty Affairs and Professional Development
2021 Associate Professor (Edu Scholar)
2022 AAMC GFA Chair-elect
2023 Wellness Coaching and Counseling Certificate
2024 LLC Career Counselor/Coach and Org Consultant
Guadalupe Federico-Martinez, PhD

## Slide 35
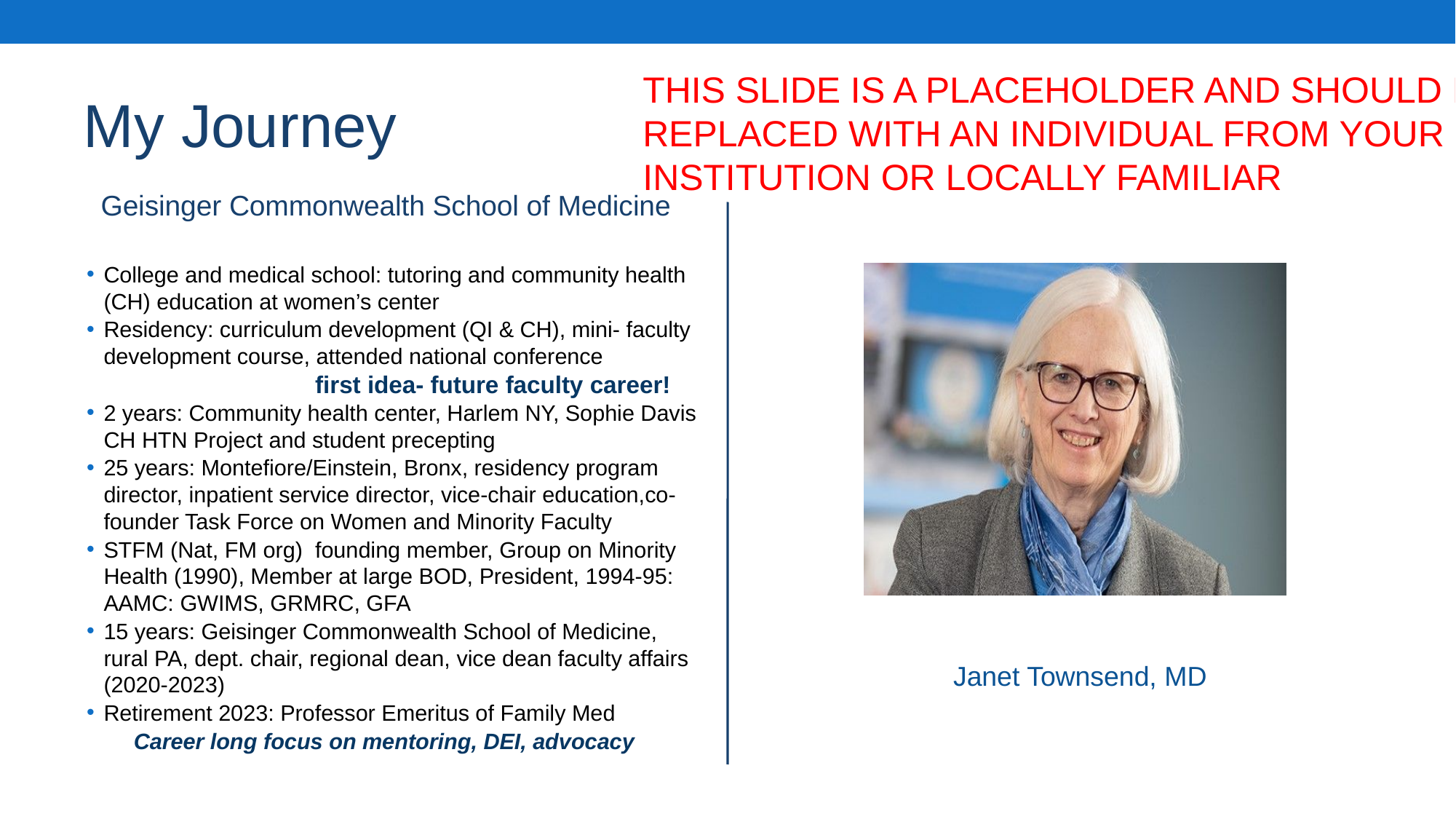

THIS SLIDE IS A PLACEHOLDER AND SHOULD BE REPLACED WITH AN INDIVIDUAL FROM YOUR INSTITUTION OR LOCALLY FAMILIAR
# My Journey
Geisinger Commonwealth School of Medicine
College and medical school: tutoring and community health (CH) education at women’s center
Residency: curriculum development (QI & CH), mini- faculty development course, attended national conference
2 years: Community health center, Harlem NY, Sophie Davis CH HTN Project and student precepting
25 years: Montefiore/Einstein, Bronx, residency program director, inpatient service director, vice-chair education,co-founder Task Force on Women and Minority Faculty
STFM (Nat, FM org) founding member, Group on Minority Health (1990), Member at large BOD, President, 1994-95: AAMC: GWIMS, GRMRC, GFA
15 years: Geisinger Commonwealth School of Medicine, rural PA, dept. chair, regional dean, vice dean faculty affairs (2020-2023)
Retirement 2023: Professor Emeritus of Family Med
Career long focus on mentoring, DEI, advocacy
first idea- future faculty career!
Janet Townsend, MD

## Slide 36
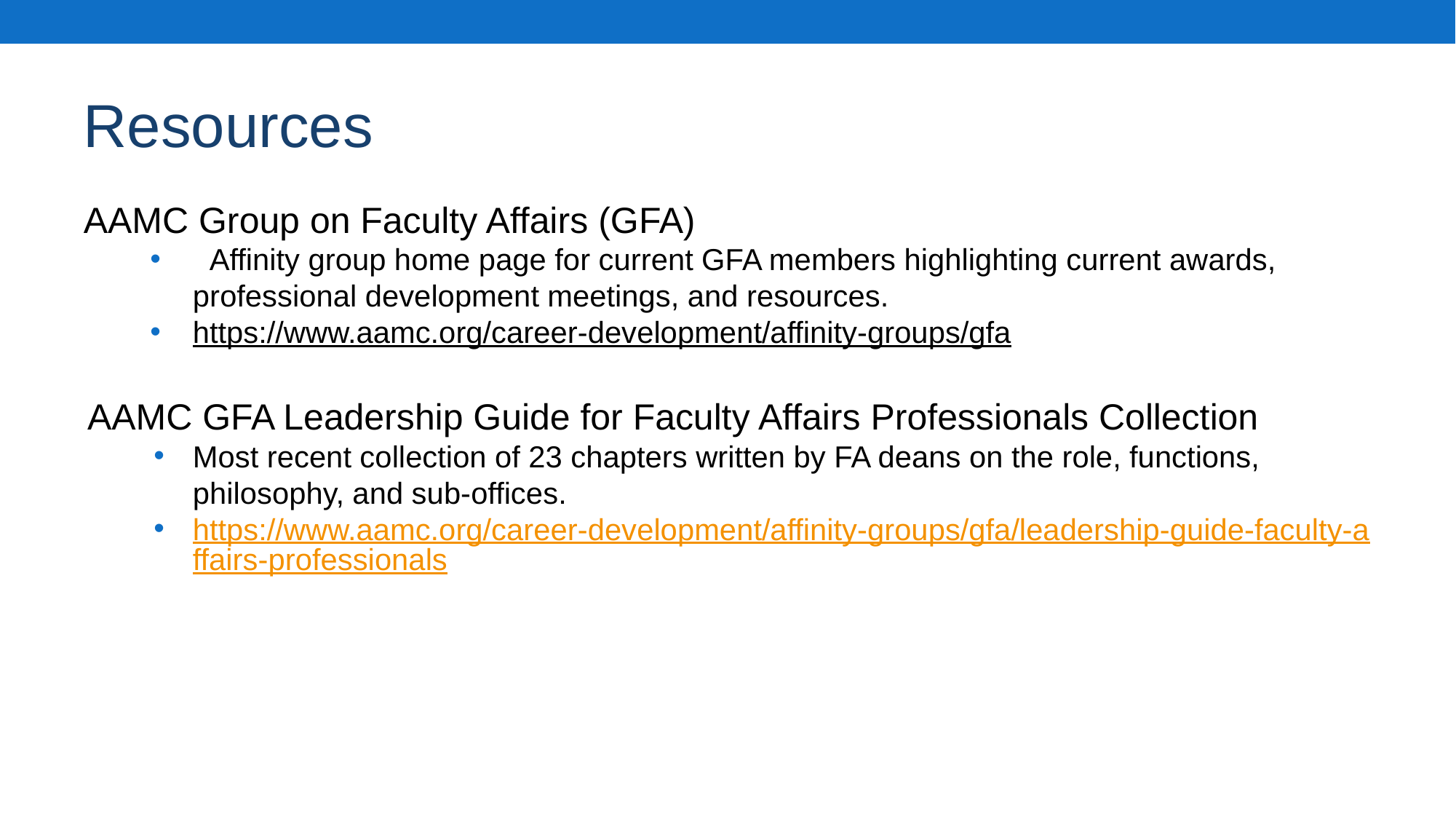

# Resources
AAMC Group on Faculty Affairs (GFA)
 Affinity group home page for current GFA members highlighting current awards, professional development meetings, and resources.
https://www.aamc.org/career-development/affinity-groups/gfa
AAMC GFA Leadership Guide for Faculty Affairs Professionals Collection
Most recent collection of 23 chapters written by FA deans on the role, functions, philosophy, and sub-offices.
https://www.aamc.org/career-development/affinity-groups/gfa/leadership-guide-faculty-affairs-professionals

## Slide 37
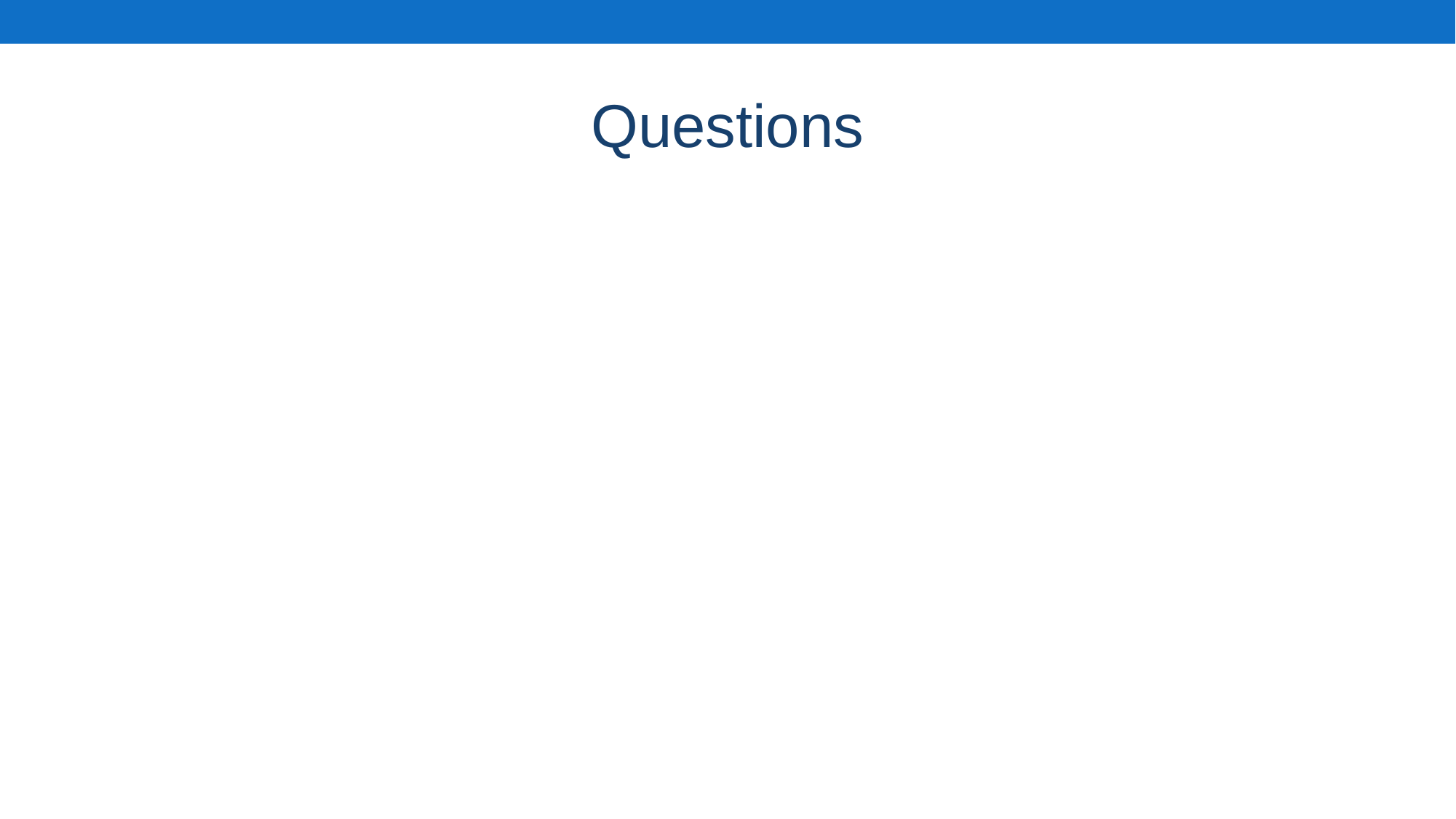

# Questions
