# Supplementary material for: Academic Career Exploration: Learner Opportunities Through the Office of Faculty Affairs
Source: MedEdPORTAL. 2024 Oct 31;20:11460. doi: 10.15766/mep_2374-8265.11460 (PMC11525038; doi:10.15766/mep_2374-8265.11460)
Supplement: Supplementary file 1 — Evaluation.docxOFA and Learner Engagement.pptxThe Value of FA and FD Offices.docxActivity Sheet.docxCase Discussion.docxExample Letter of Recommendation.docxFacilitator Guide.docx [file mep_2374-8265.11460-s001.zip › C. The Value of FA and FD Offices.docx]

This document outlines the professional tenets of faculty affairs as explained by the AAMC. This document is to be disseminated with the pre- evaluation survey.

**The Value of Faculty Affairs and Faculty Development Offices**

In academic healthcare institutions, education, research, clinical service and community engagement are essential components to the production of excellence. Faculty members are a significant force in achieving these missions, therefore, it is essential that we continue to support and invest in their career development and well-being, specifically in an environment that is constantly impacted by internal and external influences. The Faculty Affairs and Development Offices are necessary in achieving this mission.

Listed below are some of the responsibilities and duties of the faculty affairs/development offices.

**Responsibilities and duties:**

- Manage appointment, promotion, and tenure
- Provide programs to develop leadership skills
- Plan for leadership and succession
- Ensure the incorporation of diversity, equity and inclusion
- Advocate for faculty well-being and vitality
- Provide teaching, research and clinical skills
- Assist in faculty retention
- Provide and organize faculty onboarding and orientation
- Support faculty governance
- Manage the annual faculty evaluation process
- Initiate faculty reviews (post-tenure, department chair reviews, administrative reviews)
- Collaborate with human resources
- Assist faculty in Applying for awards, scholarships, fellowships and other recognition
- Provide counseling and mentoring (Mentorship Programs)
- Development of faculty handbooks, bylaws
- Collaborate with department chairs and directors to develop and standardize faculty offer letters
- Represent faculty affairs/ faculty development at the institutional level
- Collaborate with the Title IX office
- Assist with faculty leave and accommodations
- Aid faculty across the faculty career life cycle
- Review faculty policies
- Assist with clinical faculty credentialing
- Participate in institutional committees
- Assist in the faculty licensing process
- Participating in the grievance/ombudsman process
- Connecting resources to address faculty assessments
- Conduct research and assess faculty, professional and leadership programs in a scholarly way
- Uphold LCME accreditation standards for the faculty workforce
- Representation on a national level for the AAMC’s Group on Faculty Affairs
- Articulate the return on investment associated with faculty professional and leadership development
- Monitor and provide resources to manage the integration of academic health systems

Faculty Development and Faculty Affairs offices provide a centralized area for the dissemination of best practices for the development, retention and the well-being of faculty members. It is essential that these offices are provided with the highest degree of institutional support.
