# Supplementary material for: Academic Career Exploration: Learner Opportunities Through the Office of Faculty Affairs
Source: MedEdPORTAL. 2024 Oct 31;20:11460. doi: 10.15766/mep_2374-8265.11460 (PMC11525038; doi:10.15766/mep_2374-8265.11460)
Supplement: Supplementary file 1 — Evaluation.docxOFA and Learner Engagement.pptxThe Value of FA and FD Offices.docxActivity Sheet.docxCase Discussion.docxExample Letter of Recommendation.docxFacilitator Guide.docx [file mep_2374-8265.11460-s001.zip › D. Activity Sheet.docx]

This document is to be disseminated with the pre- evaluation survey and will be referenced by the PowerPoint on slides 25-28.

| **Appendix D. Activity Sheet: Office of Faculty Affairs: Engagement and Leadership Opportunities for Learners** |
| --- |

According to the 2022 AAMC Year Two Questionnaire approximately 21% of medical students plan to participate in medical school administration during their career and 60% plan to become medical school faculty.^1^ The Office of Faculty Affairs serves as one avenue for medical students to become engaged and develop competencies to serve as future administrative leaders.

**Role of the Office of Faculty Affairs:**

To oversee the recruitment, appointment, and academic promotion of faculty and senior academic leaders. Coordinates with others medical school offices, such as the Office for Diversity and Inclusion to help the school meet its diversity goals related to faculty. Oversees all policies related to faculty leave, sabbaticals, salary equity, grievances and other topics. Provides career development, guidance, and mentoring opportunities to faculty. Oversees the evaluation process for chairs and deans. Administers a system of faculty governance including faculty committees important to school operations.

**Relevance of Trainees Being Engaged in Faculty Affairs:**

Trainees may provide a critical perspective by volunteering or through selection to faculty, chair and dean search committees. Medical students are especially adept at helping candidates explore institutional fit, particularly for those underrepresented in medicine. In addition to influencing initial appointment, learners may serve on committees deciding contract renewal or promotion of senior administrators. Medical students may also serve on committees reviewing grievances against faculty members and provide a learner lens.

| Faculty Leadership Competencies Achievable Through Faculty Affairs Related Activities^2^ | | | | | |
| --- | --- | --- | --- | --- | --- |
| 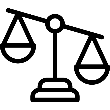  **Self-management:** demonstrates emotional intelligence in faculty searches and exercises effective time management. | | 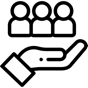  **Working with/developing others:**  strengthens interpersonal skills in discussing recruitment, (re-) appointment and promotion policies and practices | | 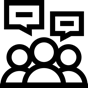  **Communication skills**: develops effective oral and written communication skills when serving on committees. | |
| 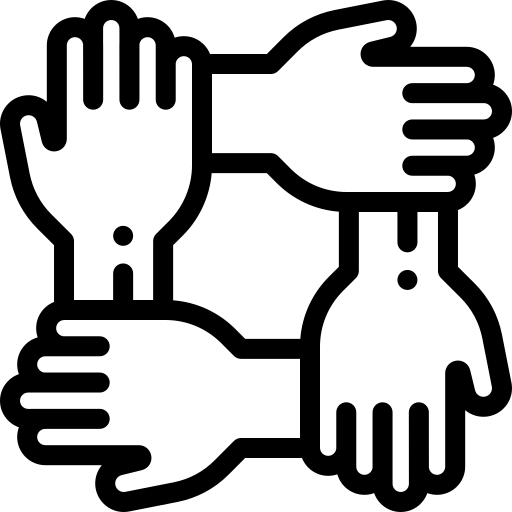    **Teambuilding**: participates in group decision making and understands power relationships when serving on search committees. | 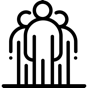  **Leadership:** understands the structure and culture of the organization. | | 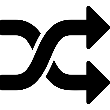  **Leading change:** understands change management through application of new or revised policies and procedures. | | 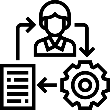 **Business skills:** understands employment packages and differences between academic roles**.** |

|  | *Types and year of participation varies by institution. | 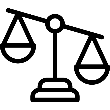 | 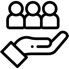 | 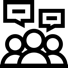 | 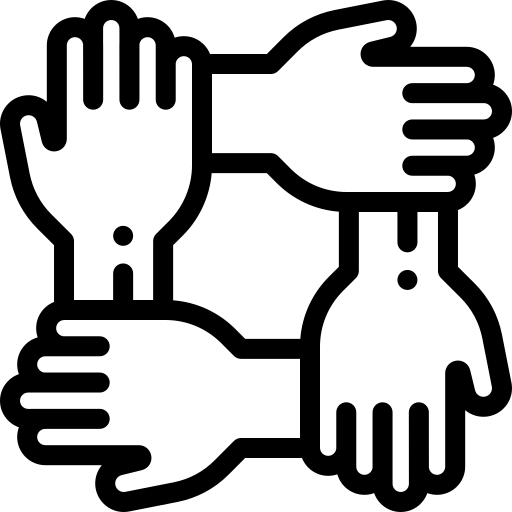 | 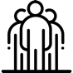 | 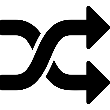 | 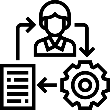 |
| --- | --- | --- | --- | --- | --- | --- | --- | --- |
| Year 1-4  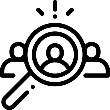 | **Serves on Faculty or Executive Search Committees**  Participates in the interviewing and provides feedback on candidates. Brings a student perspective to faculty and senior administrator recruitment. Supports the committee in identifying diverse faculty candidates. | • |  | • | • | • |  |  |
| Year 1-4  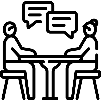 | **Serves on Standing and Ad Hoc Committees**  Participates in standing or ad hoc committees and brings the student perspective. Works with faculty and administrators to develop, revise, and apply policies and procedures, for circumstances such as re-appointment, grievances, etc. | • |  | • | • |  | • |  |
| Future  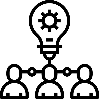 | **Leads as a Faculty Member or Administrator**  Engages in or leads efforts to support pre-faculty development - the recruitment of medical students and residents as future faculty. Aspires for a larger role as program director, committee chair, or dean of faculty affairs. | • | • | • | • | • | • | • |

**Acknowledgements:** We thank FlatIcon for the access and use of their free vector icons **References:**

1**.** Year Two Questionnaire (Y2Q). AAMC.org. Accessed on December 20, 2023. [www.aamc.org/data-reports/students-residents/report/year-two-questionnaire-y2q](http://www.aamc.org/data-reports/students-residents/report/year-two-questionnaire-y2q)

2. Lucas R, Goldman EF, Scott AR, et al. Leadership development programs at academic health centers: results of a national survey. *Acad Med*. 2018;93(2):229-236.

Author contact: J.P. Sanchez MD, MPH, BNGAP President, [bngapinc@gmail.com](mailto:bngapinc@gmail.com)

Icon made by Surang from [www.flaticon.com](http://www.flaticon.com) Free for personal and commercial purpose with attribution. <https://www.flaticon.com/free-icon/team_1124812?term=teamwork&page=1&position=26>

Icon made by Freepik from www.flaticon.com Free for personal and commercial purpose with attribution. <https://www.flaticon.com/free-icon/interview_2303881?term=interview&page=1&position=78>

Icon made by Freepik from www.flaticon.com Free for personal and commercial purpose with attribution. <https://www.flaticon.com/free-icon/recruitment_942830?term=recruit&page=1&position=51>

Icon made by Kiranshastry from [www.flaticon.com](http://www.flaticon.com) Free for personal and commercial purpose with attribution. <https://www.flaticon.com/free-icon/balance_1153269?term=balance&page=1&position=52>

Icon made by Freepik from www.flaticon.com Free for personal and commercial purpose with attribution. <https://www.flaticon.com/free-icon/teamwork_921332>

Icon made by Freepik from www.flaticon.com Free for personal and commercial purpose with attribution. <https://www.flaticon.com/free-icon/target_1605401?term=support&page=1&position=8>

Icon made by Those Icons from www.flaticon.com Free for personal and commercial purpose with attribution. <https://www.flaticon.com/free-icon/shuffle_2089813?term=change&page=1&position=44>

Icon made by Eucalyp from www.flaticon.com Free for personal and commercial purpose with attribution. <https://www.flaticon.com/free-icon/process_1556324>

Icon made by Freepik from www.flaticon.com Free for personal and commercial purpose with attribution. <https://www.flaticon.com/free-icon/group_909337>

Icon made by Freepik from www.flaticon.com Free for personal and commercial purpose with attribution. <https://www.flaticon.com/free-icon/team_478536>
