# Supplementary material for: Academic Career Exploration: Learner Opportunities Through the Office of Faculty Affairs
Source: MedEdPORTAL. 2024 Oct 31;20:11460. doi: 10.15766/mep_2374-8265.11460 (PMC11525038; doi:10.15766/mep_2374-8265.11460)
Supplement: Supplementary file 1 — Evaluation.docxOFA and Learner Engagement.pptxThe Value of FA and FD Offices.docxActivity Sheet.docxCase Discussion.docxExample Letter of Recommendation.docxFacilitator Guide.docx [file mep_2374-8265.11460-s001.zip › E. Case Discussion.docx]

This document is to be disseminated with the pre- evaluation survey and will be referenced by the PowerPoint on slides 30-32.

Appendix E.

Case 1

Lola is a 2nd-year medical student and has been asked to serve on the search committee for the new Diversity, Equity, and Inclusion Director of her osteopathic medical school. The search is led by the Office for Faculty Affairs and Career Development. The committee will consist of volunteer members from across the medical school, including staff, faculty, and learners. As a part of the search committee, Lola will participate in biweekly meetings to review applications, interview candidates, and determine the top three candidates for the Dean to consider.

The candidates will be reviewed for their background and experiences and present their philosophy/approach to leading initiatives in DEI, including addressing systemic issues related to programming, recruiting, retaining, and supporting diverse faculty, staff, and students, and addressing bias complaints. All committee members will be asked to attend a session on holistic review and anti-bias training.

- How should Lola prepare for this committee work?
- What leadership competencies will Lola develop by participating on this committee?
- Can Lola document this committee work on her CV?
- Lola greatly enjoys the experience and wonders what future opportunities through the Office for Faculty Affairs and Career Development she can participate in?

Case 2.

Maria is a 1^st^ year medical student and just learned that she received the top grade in her anatomy class. She has great admiration for her anatomy professor, Dr. Martinez, who she credits with helping her succeed. She considers Dr. Martinez to be an excellent lecturer, who provides information clearly and reviews content with students repeatedly to ensure they understand core concepts.

Dr. Martinez approaches Maria to ask her if she would be willing to write a letter of recommendation for promotion from Assistant to Associate Professor. Maria has never written a letter of recommendation for faculty promotion and wants to make sure she writes a thoughtful and supportive letter for her professor.

- How should Maria prepare for writing this recommendation letter?
- What leadership competencies will Maria develop by writing this recommendation letter?
- Can Maria document this work on her CV?
- Maria greatly enjoys the experience and wonders what future opportunities through the Office for Faculty Affairs and Career Development she can participate in?

Case 3.

Jaime is a 4^th^ year medical student and has been asked by the Office for Faculty Affairs and Career Development (OFACD) to participate on the Faculty Grievance Advisory Committee. The committee recently received statements from 3 medical students regarding the professionalism of a faculty member. The student has been asked to provide the perspective of the medical student as this and other cases are reviewed.

The committee consists of OFACD Deans, 2 departmental chairs, and 2 full professors. All committee members are expected to participate in monthly meetings, review complaints, and provide their perspective on the case.

- How should Jaime prepare for this committee work? What concerns may Jaime have and how can she explore them?
- What leadership competencies will Jaime develop by participating on this committee?
- Can Jaime document this committee work on their CV?
- Jaime greatly enjoys the experience and wonders what future opportunities through the Office for Faculty Affairs and Career Development he can participate in?
