# Supplementary material for: Academic Career Exploration: Learner Opportunities Through the Office of Faculty Affairs
Source: MedEdPORTAL. 2024 Oct 31;20:11460. doi: 10.15766/mep_2374-8265.11460 (PMC11525038; doi:10.15766/mep_2374-8265.11460)
Supplement: Supplementary file 1 — Evaluation.docxOFA and Learner Engagement.pptxThe Value of FA and FD Offices.docxActivity Sheet.docxCase Discussion.docxExample Letter of Recommendation.docxFacilitator Guide.docx [file mep_2374-8265.11460-s001.zip › F. Example of Letter of Recommendation.docx]

The letter below is an example of a successful letter of support for tenure by one of the authors of the module.

**Letter of Support for Tenure Application for Dr. XXXX**

To XXXX

I am writing this letter on behalf of my mentor Dr. XXXX, whom I have had the absolute privilege and honor of knowing over the last three years. Dr. XXXX has guided my academic, professional, and personal growth from the completion of my undergraduate studies, successful matriculation into medical school, and to my first submitted publication to MedEdPORTAL.

By chance, one afternoon on an escalator to class, I met Dr. XXXX for the first time at a college medical school information session. Enthusiastically, he asked me if I was interested in academic medicine. As a senior pre-medical student, I was not familiar with the term nor what it entailed. In fact, I had insecurities about being accepted into medical school and how I would perform on the Medical College Admissions Test (MCAT). I was the first in my family to pursue a STEM degree and aspire to become a physician. But, at that moment, I trusted Dr. XXXX as his deep passion for the topic persuaded me to follow up. Truthfully, I was lost in the process of applying to medical school and intimidated as a black male with a dream of entering medicine and a goal of giving back to my community.

I can attribute my success of multiple medical school acceptances, scholarship offers, research project opportunities, and first submitted publication to the guidance of Dr. XXXX. He was able to materialize my own passion and purpose through avenues I would not have discovered if it were not for his mentorship. Dr. XXXX guided me through the medical school application process which allowed me to discover my own competitiveness and self-worth. His investment in reading over my applications, holding mock interviews, MCAT strategizing, and motivating advice reinforced my purpose for pursuing medicine. He introduced me to Building the Next Generation of Academic Physicians (BNGAP), an organization focused on pre-faculty development for medical trainees. I attended workshops where I discovered how my interest in serving marginalized communities could be translated into scholarship and make an impact on a systematic level. Also, Dr. XXXX introduced me to his network of colleagues where I learned the importance of fostering a strong teamwork environment and taught me how to effectively coordinate conferences and national meetings. In failure, he provides reassurance that empowers me to reface challenges and in success, he pushes me to accomplish more. Dr. XXXX set the spark that kindled my own discovered interest to become a future clinician and academician.

With that said, I am highly confident in my support for Dr. XXXX. As a future physician and hopeful faculty member, I wish to embody the same admirable characteristics that Dr. XXXX possesses. I look forward to Dr. XXXX’s continued guidance and mentorship and I am excited for the meaningful work that he will help me accomplish.

If you would like to speak further about my experience working with Dr. XXXX, please feel free to reach me at [XXXXXX@XXX.XXX](mailto:XXXXXX@XXX.XXX) or by cell phone at XXX-XXX-XXXX.

Sincerely,

**XXXX**

Medical Student
