# Supplementary material for: Academic Career Exploration: Learner Opportunities Through the Office of Faculty Affairs
Source: MedEdPORTAL. 2024 Oct 31;20:11460. doi: 10.15766/mep_2374-8265.11460 (PMC11525038; doi:10.15766/mep_2374-8265.11460)
Supplement: Supplementary file 1 — Evaluation.docxOFA and Learner Engagement.pptxThe Value of FA and FD Offices.docxActivity Sheet.docxCase Discussion.docxExample Letter of Recommendation.docxFacilitator Guide.docx [file mep_2374-8265.11460-s001.zip › G. Facilitator Guide.docx]

The facilitator guide is to be used by the speakers to guide the presentation. We recommend prospective speakers to review this document before the presentation.

**Facilitator Guide**

**Overall Goal**

The overall goal of the workshop is to raise medical student awareness of the structure and function of offices of faculty affairs and opportunities to become engaged in the office during medical school or residency.

**Learning Objectives**

- Describe the ABCs of Offices of Faculty Affairs;
- List skills and characteristics needed to work in faculty affairs;
- Describe learner involvement in faculty affairs

**Timetable (60 minute presentation)**

00:00-03:00 minutes Pre-assessment

04:00-12:00 minutes Introduction and Reflection – slide 1-4

13:00-30:00 minutes PPT presentation slides 5-28

31:00-45:00 minutes Case discussion – 29-32

46:00-57:00 minutes Facilitator journey and questions 33-37 slides

58:00-60:00 minutes Post-assessment

**Materials**

Computer set-up for PPT presentation

Print out pre- and post-assessment materials

Print out cases

Pen or pencils for students to take notes on case questions

**Slide Instructions**

Slide 1

The facilitator should introduce themselves, including their role or relationship with the office of faculty affairs.

Slide 2

Review learning objectives with workshop participants.

Slide 3

Review session highlighting the various interactive components.

Slide 4

This exercise is to help engage the audience and precipitate dialogue. Listen and learn about learners’ experiences with the Office for Faculty Affairs. Learners should be aware that it may also be referred to as Office for Faculty Affairs and Career Development.

Reflection:

Does your school have an Office of Faculty Affairs?

*It is important to mention that most medical schools have a distinct Office of Faculty Affairs.*

Have you ever walked into the Office of Faculty Affairs? If yes, why?

*It is important to note that most medical schools have a physical space and webpage of the Office of Faculty Affairs.*

Have you ever received a lecture or presentation from the Office of Faculty Affairs? Ask how do the faculty know that the person they interacted with was from the office of faculty affairs?

*It is important to note that most learners’ have not received a lecture or presentation from the Office of Faculty Affairs, in part because the office of faculty affairs focuses on faculty. However there are opportunities for learners’ to be engaged in the work of OFA*

Slide 5

Remind viewers that this is a typical structure, but that every institution is unique, with some institutions having the president of the system and the dean being the same person. In most medical schools, there is a dean position devoted to faculty affairs, or sometimes faculty and academic affairs. The position typically reports directly to the Dean

Slide 6

Insert a slide of the organizational chart of your school, and point out where faculty affairs are situated.

Slide 7

Institutions are expected to involve faculty members in a "shared governance" model of administration where both faculty and administrators of the school give input and take responsibility for the various mission areas of the school. This is typically carried out through faculty committees that advise the dean and medical school administration on various mission areas within the school - curriculum, education, research, etc. While a certain dean (education, research, etc.) may be the main administrative link for these committees, OFA is the office that typically will oversee the process of ensuring broad faculty participation in these committees, ensuring they run smoothly, and that the shared governance model is effective.

Individual institutions may have additional governance such as faculty councils that may be subject to oversight by university faculty councils.

WHY is this important? Many schools reserve spots for STUDENT members on these committees which is one of the main ways you have opportunities to interact with OFA.

Slide 8

Review common mission and goals of OFA. Of these missions of OFA, faculty development is often one that helps the school achieve its mission and make improvements. For example, if the curriculum committee recommends increasing team based learning in the classroom there needs to be faculty development to ensure faculty have the skills to do that. If a priority of the Dean or University President is to increase research output, OFA may implement faculty development to enhance faculty research skills, or to plan to hire new faculty with these skills.

Slide 9

In addition to the larger missions mentioned, OFA often has very discreet responsibilities related to all of the “HR” type of functions for faculty members. Review common duties and responsibilities of OFA.

Appendix C includes common duties and responsibilities as described by AAMC GFA. GFA Steering Committee: Lisa Cain, Guadalupe Federico-Martinez, Elza Mylona, Michelle Oliva, Michele Kutzler, Johnson George, Sheila Crow, Jennifer Apps. Prepared for AAMC Council on Deans orientation. June 2023.

Slide 10

Transition slide. Explain to learners that you will focus on three components of OFA leaders – 1) administrator and advocate; 2) Basics, behavior, and benefits; and 3) Communication, confidentiality, and culture

Slide 11

Self-explanatory.

Leaves can be sabbaticals, or other extended absences. Advocacy is empowered by the faculty councils, and OFA can use the faculty councils as the advisory group for the office.

Slide 12

Self-explanatory.

Slide 13

Self-explanatory

Slide 14

Faculty have an opportunity to be promoted and attain different ranks and titles based on their achievements. Faculty rank is both an internal and external way of sharing that one has succeeded to a certain level as a faculty member. Typically, these ranks start at the level of Instructor, and then move up to the level of full Professor. OFA is typically responsible for administering this process of faculty academic promotion.

Slide 15

In addition to higher academic ranks, in many schools faculty can achieve TENURE. Tenure is typically reserved for faculty members with such exemplary accomplishments that they are given a permanent faculty position in the school. Historically, tenure was an important tool that allowed for academic freedom, as faculty sometimes voice opinions, author works, or conduct research in controversial areas. Tenure gives them the protection of not having their job at risk for doing this. Even today, when things such as critical race theory, reproductive rights, and gender affirming care have become politicized, faculty who study or engage in these areas have a level of job protection afforded by achieving tenure.

Malone W. and Cox N. Trends in Faculty Appointment, Promotion, and Tenure Policies and Practices in the U.S. Medical Schools. June 2023.

Slide 16

One of the main duties of OFA is to oversee the process for academic promotion and tenure. This typically involves giving guidance to faculty to create a “promotion dossier” that describes their achievements in scholarship or research, teaching and service. OFA typically works with the Promotions Committee, made up of peer faculty, who evaluate these dossiers and make recommendations to the university for academic promotion and tenure.

Slide 17

At most medical schools, faculty choose or are hired into a “track” which describes the academic areas of focus for their career. Tracks usually fall within the categories of research, education and clinical service. In some schools, tracks could also include areas of emphasis such as community engagement. This helps align the faculty members interests with their job duties and with the criteria that will be used to evaluate them for academic promotion or tenure.

Slide 18

Transition Slide

Now that we have discussed what the Office of Faculty Affairs does, the following are some ways that you can improve your leadership skills and engage, as a medical student or resident, with OFA.

Slide 19

Learners are often asked to write a letter of recommendation for a faculty member for their initial faculty appointment or subsequent promotion. Appendix F provides an example of a letter.

**Writing a Letter of Recommendation (LOR) for Faculty Promotion**

- Stress why LORs are important to faculty promotion just as evaluations are.
- Offer the idea that writing such narratives and engaging in deep reflection about the professional relationship one has with another prepares them for all the LORs they will be asked to do as a faculty member or FA leader in the future.
- Emphasize that general **LOR best practices** tend to focus on the faculty member writing for a student and not the other way around, however, the general principles for a compelling letter can be applied. As an aside, briefly state the capacity and length in which you worked with them; the exact title, track, and aspiring rank, and be mindful of mitigating bias language in your draft.
- If there is time you can suggest what a student could **plan for** if solicited. Below are some points you can draw from and will be unique to each request/site.
- A 1-3 month turn around time to return the letter by a deadline
- Spending no less than 3 hours to reflect, and research their profile or have a conversation if you have questions or refreshment of memory.
- A formal solicitation and submission protocol that OFA or the candidate will communicate.
- Perusal of best practices or FAQ in letter writing on your OFA or OSA website.
- Perusal of promotion criteria for their specific track and rank.

Reference: Dunnick NR. Letters of recommendation for promotion. *Academic Radiology*. 2022;29(1):1-3.

Slide 20

Self-explanatory. Typical promotion or tenure process. Early on department or OFA solicit letters from learner(s) for packet

Slide 21

**What is Faculty Grievance?**

Include terms or nomenclature common to your site. Grievance in OFA can also include terms such as Conflict Resolution Process, Professionalism and Mistreatment Process or Compliant.

Common as part of the faculty bylaws. A faculty ombudsperson with a certificate in conflict resolution, third party mediator (JD), or executive coach may lead informal or formal hearings. As such, emphasize which is the common structure at your site and why (i.e., SOM faculty governance policies, private institution or board of regents/state laws, etc.). Comment on the inclusion or exclusion of medical students or residents on the clearinghouse or committee. Do they sit with the general faculty, do they get a vote, why are or aren’t they permitted to serve as a representative to date?

Slide 22

**Typical Grievance Process**

The typical grievance process initiates with the faculty member filing a written complaint that lists the reason. It is important to stress that the process and scope of the process is applicable to faculty who are paid employees of the university system and not the volunteer/adjunct or hospital paid faculty unless your site has a rare exception to the rule. Also, relate the initiation process and recommendation for conciliation starting at the division/dept/dean level before escalating to the university. In other words, does your process encourage working your way informally first and up the chain?

Early on learners are sometimes invited to serve on committee.

Facilitators can adapt the slide to reflect your site.

Slide 23

**What is a Search Process?**

OFA facilitates the executive recruitment process. Detail out the below functions for the learner by each stage *in the way and to the extent that they play out at your site*:

Pre-Search

1. OFA can use either internal recruiter, HR partner, or engage with a corporate search firm to source and outreach national/international prospects. May also work with the Office of Diversity, Equity and Inclusion to ensure outreach to a rich pool of diverse candidates, to mitigate implicit bias in the search process, and promote an inclusive onboarding process.
2. Works with the dean and immediate supervisor-to-be to solidify position description and committee composition

Search

1. Facilitates institutionally required training, interview protocols, confidentiality agreements, scoring rubrics, candidate debriefs, itineraries for site visits.
2. Provides support and education to the search committee chair on policy and procedures.
3. Key liaison between committee, firm, candidate finalist, and the dean.
4. Students are invited to participate as committee members or interviewers for follow-up visits.
5. Co-draft Offer Letters with the dean and HR. Advise on employment chapter, professorial track and rank as appropriate.

Post-Search

1. Ensure new leader/faculty orientation and onboarding.
2. Guide the role’s transition team at arm's length.
3. Connect to (and remunerate) executive coaching as requested or supported by the school, or clinical home department.

Slide 24

**Typical Search Process**

- A Typical search process might involve the process articulated on the slide. Again, each site varies, and the engagement with a search firm will augment the process moderately, and the extent to which OFA/HR and students are involved. Adapt the graphic to reflect your site.
- Learners are invited to serve on committee

Slide 25

Read list of leadership development needs for faculty affairs

Slide 26

Skills and characteristics. Tie back to the ABC’s of faculty affairs discussed earlier in the presentation

Slide 27

These are the seven leadership competencies of importance for faculty success. Several of these can be supported by working with the Office of Faculty Affairs, primarily through being student members on committees

Slide 28

Potential Learner Involvement

These are examples of opportunities through OFAs that incorporate more trainee perspectives. Facilitators can review these examples as potential opportunities for their own site to engage in if they do not already do them. Facilitators are encouraged to augment the slide to reflect additional/different points.

Slide 29

Transition slide

This is an interactive exercise for learners to apply their knowledge of the Office of Faculty Affairs and faculty development competencies in discussing cases. Learners should be broken into one of 3 groups. In 10 minutes they should discuss the case and associated questions in a small group and then return to a large group discussion and have one person per case read case and discuss responses to the questions.

Slide 30

| Question | Response |
| --- | --- |
| - How should Lola prepare for this committee work? | - Start thinking of what she would value in a DEI director - Research different directors and what people should consider when assuming this position. - Think about the qualities of leaders she values to look for in the candidates. |
| - What leadership competencies will Lola develop by participating on this committee? | - Self-management was developed by contributing reflections to the search committee in assessing candidates for position, including time management demonstrated in timely responses. - Working with/developing other was developed by participating in the practice of appointing a new director in the medical school - Communication skills was developed by writing emails to rank candidates with explanations of strengths and weaknesses associated with expectations for the role - Team building was developed by working with directors, deans, faculty, staff and other medical students in the process of finding a new DEI director - Leadership was demonstrated by understanding and contributing to promoting the culture of the medical school with the hiring of a new director leader |
| - Can Lola document this committee work on CV? | - Placed under the title of Extracurricular activity, Committee work & Leadership on her CV - Experience type (that could also be written on an ERAS application): education/training, specifically, training on how to go through a hiring process |
| - Lola greatly enjoys the experience and wonders what future opportunities through the Office for Faculty Affairs and Career Development she can participate in? | - She can be involved in a number of OFA initiatives focused on evaluating and improving OFA efforts, including faculty recruitment, retention and development practices. Her experience in the recruitment process would also help prepare her to get involved in faculty grievance processes. |

Slide 31

| Question | Response |
| --- | --- |
| - How should Maria prepare for writing this recommendation letter? | Review the literature on how to write a compelling quality letter of recommendation. Also refer to the following:   - Includes but goes beyond teaching, research, clinical skills and knowledge. - Provides insight into their character, role modeling, commitment to mentoring of you and shared accomplishments. - Reads as a compelling narrative/story that gets at emotions as opposed to reciting their CV. - Explicitly, articulate your unwavering support. |
| - What leadership competencies will Maria develop by writing this recommendation letter? | - Self management – critically considers how the faculty member has supported their own personal, professional and academic development and how they may be an asset to others the institution - Communication skills – critically considers how to construct a letter that details how the faculty member has met the criteria for promotion - Leadership – understands the needs of their peers and strives to promote faculty who are dedicated to supporting the well-being of other learners |
| - Can Maria document this work on her CV? | Maria should review the CV templates offered through her school. Often there is a section where individuals can note letters of recommendation written for others (e.g. for faculty to be promoted; pre-medical students application to medical school; etc.) If you are unsure how to add to CV also inquire with your office of faculty affairs |
| - Maria greatly enjoys the experience and wonders what future opportunities through the Office for Faculty Affairs and Career Development she can participate in? | Maria can sit with the leadership of OFA and inquire of opportunities. Some other opportunities may be participating on search committees and grievance committees. |

Slide 32

| Question | Response |
| --- | --- |
| - How should Jaime prepare for this committee work? | Immediately upon invitation, Jaime should review the Faculty Affairs website to review public information on the description of the committee, policies, resources, and process ahead of time.  Even before completing formal training, practice self-management by only disclosing upcoming participation dates to his advisor and relevant others. Similar to IRB (or CITI) or HIPAA/PHI training about confidentiality, privacy, and conflict of interest, Jaime can begin considering his part in the importance of protecting the information and people involved in the pending case.  Work with the grievance committee chair (or surrogate) to ensure all required orientation and training modules are complete before the first charge meeting. He can expect topics to include:  *Note: This list is not exhaustive and varies by site.*   - Implicit Bias Training - Maintaining psychological safety and non-judgmental non-verbal/verbal communication. - Compassionate curiosity - Confidentiality/privacy - Conflict of Interest - Deliberation rubric - Policies, conflict management resources, and processes   Mental and emotional preparation should include an understanding that Jaime does not need to be an expert in mediation or conflict resolution to be an effective participant. Rather, Jaime can understand that he will bring a much-needed student perspective and use the experience to familiarize himself with available conflict management resources and approaches. This is a prized opportunity to practice necessary leadership skills in preparation for faculty life.  Manage his calendar/time by blocking out small segments of time reserved for the monthly meetings, and document review of the complaints. |
| - What leadership competencies will Jaime develop by participating on this committee? | **Self-management**: Will be developed by Jaime’s application of the training modules to hear cases/mediate/participate in deliberation. Most training modules on grievance give rise to the principles of emotional intelligence.  **Teambuilding:** Will be developed through his first-hand witnessing of power dynamics between faculty-to-faculty or faculty-to-student -or faculty-to-mediator. Additionally, Jaime will have direct participation in active listening to the issue from multiple perspectives, discussion, a collective decision or recommendation for a resolution.  **Working/developing others**: Will be developed by Jaime having the opportunity to engage in interpersonal thought exchange with the committee and faculty member as the case moves through discovery and resolution phases. He will play a role in the development and growth of everyone involved.  **Communication skills**: Will be developed by Jaime engaging in written, oral, and body language communication throughout the process. |
| - Can Jaime document this committee work on his CV? | Yes, under college/school of medicine level - service/outreach committee work. His role can be listed as “member” or “medical student representative” depending on what is customary at the institution. |
| - Jaime greatly enjoys the experience and wonders what future opportunities through the Office for Faculty Affairs and Career Development he can participate in? | At the end of the experience, Jaime could send a “thank you” email to the chair and include the OFA director and/or deans to express his desire to remain involved in upcoming opportunities that might engage students.  Jaime could offer time once or twice an academic year to serve as a student representative on Executive Leadership searches for the college/school. Another opportunity could be serving as a student representative as part of focus group interviews for specific 5–7-year administrator reviews of the dean or individual Vice/Sr. associate deans within the dean’s leadership cabinet. |

Slide 33 - 35 These are examples of career journeys for three faculty affairs deans. Ideally the presenter will use these as a model, and insert a slide of their own journey. If the presenter is not a faculty affairs professional, use one or all of these as examples of how one can progress in their career towards a career in faculty affairs.

Slide 36

Self-explanatory

Slide 37

End the presentation by asking the audience if they have any questions or reflections on what they learned today.
